# Compensation of Speckle Noise in 2D Images from Triangulation Laser Profile Sensors Using Local Column Median Vectors with an Application in a Quality Control System

**DOI:** 10.3390/s25113426

**Published:** 2025-05-29

**Authors:** Paweł Rotter, Dawid Knapik, Maciej Klemiato, Maciej Rosół, Grzegorz Putynkowski

**Affiliations:** 1 AGH University of Krakow, 30-059 Krakow, Poland; knapik@agh.edu.pl (D.K.); mkl@agh.edu.pl (M.K.); mr@agh.edu.pl (M.R.); 2 CBRTP S.A. (Centrum Badań i Rozwoju Technologii dla Przemysłu S.A.), 00-645 Warszawa, Poland; grzegorz.putynkowski@cbrtp.pl

**Keywords:** laser profilometer, speckle noise, column fixed pattern noise, optical inspection

## Abstract

The main function of triangulation-based laser profile sensors—also referred to as laser profilometers or profilers—is the three-dimensional scanning of moving objects using laser triangulation. In addition to capturing 3D data, these profilometers simultaneously generate grayscale images of the scanned objects. However, the quality of these images is often degraded due to interference of the laser light, manifesting as speckle noise. In profilometer images, this noise typically appears as vertical stripes. Unlike the column fixed pattern noise commonly observed in TDI CMOS cameras, the positions of these stripes are not stationary. Consequently, conventional algorithms for removing fixed pattern noise yield unsatisfactory results when applied to profilometer images. In this article, we propose an effective method for suppressing speckle noise in profilometer images of flat surfaces, based on local column median vectors. The method was evaluated across a variety of surface types and compared against existing approaches using several metrics, including the standard deviation of the column mean vector (SDCMV), frequency spectrum analysis, and standard image quality assessment measures. Our results demonstrate a substantial improvement in reducing column speckle noise: the SDCMV value achieved with our method is 2.5 to 5 times lower than that obtained using global column median values, and the root mean square (RMS) of the frequency spectrum in the noise-relevant region is reduced by nearly an order of magnitude. General image quality metrics also indicate moderate enhancement: peak signal-to-noise ratio (PSNR) increased by 2.12 dB, and the structural similarity index (SSIM) improved from 0.929 to 0.953. The primary limitation of the proposed method is its applicability only to flat surfaces. Nonetheless, we successfully implemented it in an optical inspection system for the furniture industry, where the post-processed image quality was sufficient to detect surface defects as small as 0.1 mm.

## 1. Introduction

**Figure 1:**
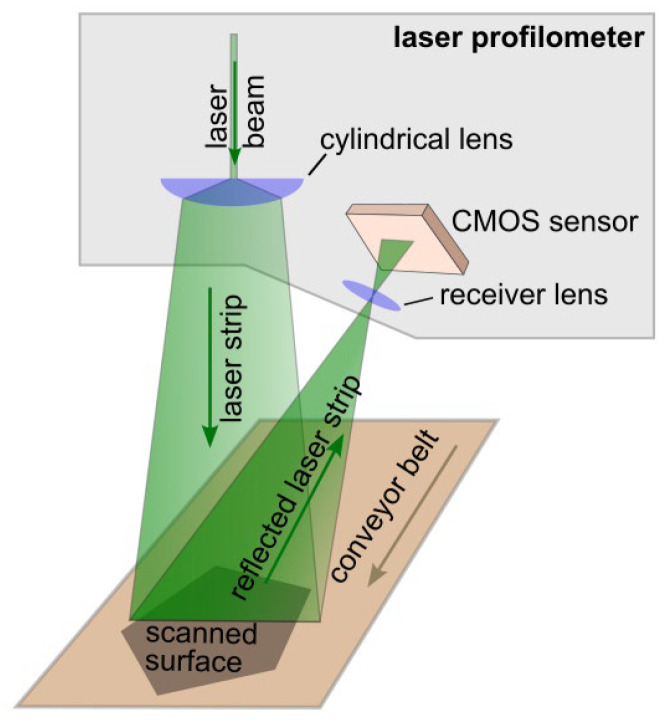
Working principle of a laser profilometer.

The triangulation laser profile sensor—also known as a laser profilometer [1], laser profiler, or line laser sensor—is a device designed for non-contact 3D scanning of moving surfaces [2,3]. It operates based on the principle of laser triangulation to determine the distance to an object. The working principle is illustrated in Figure 1. A laser beam is shaped into a line using a system of cylindrical lenses. This line is projected onto the object’s surface, and the reflected light is captured by a high-resolution CMOS camera integrated into the profilometer. The position of the reflected laser line on the camera’s sensor enables the calculation of the angle between the projected and reflected beams. This, in turn, allows for the precise determination of the distance from the sensor to each illuminated point on the object’s surface. As the object moves along a conveyor belt in a direction perpendicular to the laser line, a complete surface scan is progressively acquired. This enables the reconstruction of the 3D coordinates for each point on the scanned surface.

**Figure 2:**
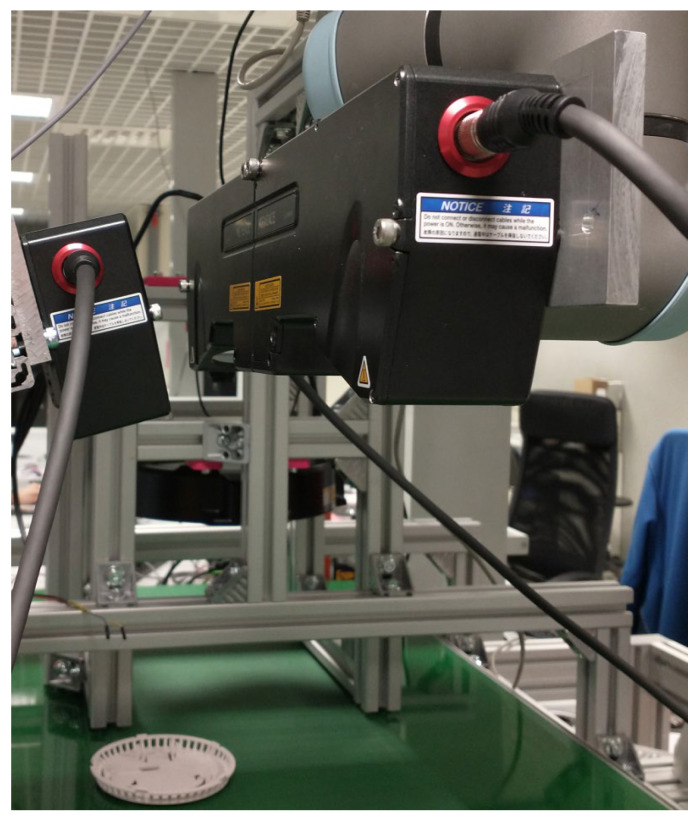
Set of four profilometers in our laboratory stand for 3D object scanning.

Such scans can be used to assess surface properties such as waviness and roughness [4], as well as to detect surface defects [5]. Additionally, they enable the construction of a 3D model of the object, particularly when multiple profilometers are used simultaneously. However, this technique has some limitations. It requires that the scanned surfaces are non-specular, as mirror-like reflections can prevent the sensor from detecting the reflected laser beam. Furthermore, the geometric features of the measured objects may cause occlusions of the laser line, restricting the completeness of the scan. These limitations can be mitigated by employing multiple profilometers positioned at different angles. Our laboratory system is an example of this approach, where a set of profilometers is used to construct a 3D model of one side of an object as part of an optical quality control system, as shown in Figure 2.

While three-dimensional imaging provides precise information about an object’s geometry, many types of surface defects can be effectively detected using two-dimensional (2D) images. In fact, most defect detection methods developed over recent decades rely primarily on 2D data [6]. As a result, many optical inspection systems based on 3D scanning also include analysis of 2D luminance images of the surface. For example, quality control of furniture boards typically involves both 3D inspection to identify shape defects and 2D image analysis to assess visual surface quality.

These two inspection tasks can be performed at separate stations—one using a profilometer for 3D scanning and the other using a conventional camera for image capture [7]. However, since a CMOS sensor is already integrated into the profilometer, many devices are capable of simultaneously acquiring grayscale images of the moving surface during 3D scanning. This opens the possibility of consolidating both inspection functions into a single unit.

The main limitation preventing the use of the profilometer as both a 3D scanner and a grayscale imaging device is the relatively low quality of the luminance images it captures. This degradation is mainly due to the inherent properties of laser light. In profilometers, the laser beam is expanded along the X-axis to form a line. Although the light waves across the X-axis share the same frequency, they exhibit varying phases. This spatial phase variation leads to interference effects, resulting in a random pattern of brightness fluctuations known as speckle noise.

Speckle noise is a well-known phenomenon that poses a significant limitation across various imaging methods, including optical coherence tomography (OCT), synthetic aperture radar (SAR), and ultrasound imaging. Numerous hardware and software-based approaches have been proposed for its mitigation; a comprehensive overview is provided in [8]. In recent years, machine learning techniques have gained prominence in this domain. Examples include the application of convolutional neural networks [9], reinforcement learning for SAR image denoising [10], and denoising diffusion probabilistic models for despeckling OCT images [11]. Nevertheless, classical signal processing methods remain viable alternatives. For instance, in [12], discrete wavelet transform-based algorithms were used for ultrasound image denoising, while [13] introduced an adaptive fuzzy filtering method for speckle reduction in SAR imagery.

Substantial research efforts have also focused on enhancing image quality from widely available imaging devices, such as smartphones and DSLR cameras. Particularly active progress has been observed in machine learning-based methods for denoising consumer-grade images, addressing issues such as low-light noise, Gaussian noise, and compression artifacts, as demonstrated in recent works [14,15].

However, direct comparisons between these approaches and those designed for profilometer image denoising are not appropriate due to fundamental differences in both noise characteristics and application requirements. Profilometer images are affected by structured, directionally correlated noise patterns—most notably, column-like speckle noise resulting from laser light interference. Unlike the statistically independent noise typically modeled in consumer imaging, or in techniques such as OCT, SAR, and ultrasound, speckle noise in profilometer images exhibits strong spatial dependencies.

Moreover, the performance metrics and visual quality criteria commonly used in general photography and medical imaging may not align with the demands of industrial inspection. In such contexts, even submillimeter deviations in surface representation can be critical for accurate defect detection. Therefore, specialized denoising techniques and evaluation methods are required to address the unique challenges posed by profilometer image processing.

**Figure 3:**
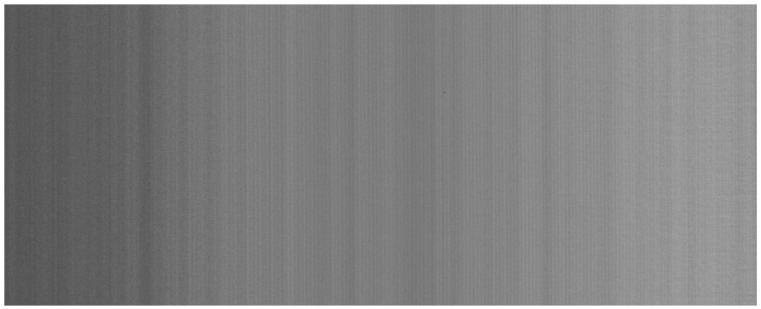
Image of a flat homogeneous surface captured by profilometer.

In intensity images captured by profilometers, speckle noise exhibits a characteristic pattern, typically appearing as vertical stripes (Figure 3). This occurs because the grayscale image acquired alongside the 3D scan is constructed line-by-line, in a manner analogous to that of a line-scan camera. As a result, the noise resembles column fixed pattern noise (CFPN) observed in CMOS frame cameras [16,17,18] and is even more visible in time-delay integration (TDI) line-scan CMOS cameras [19].

However, the column speckle noise (CSN) observed in profilometer images differs from conventional CFPN in several important ways. CSN is generally stronger and more variable: the position of the noise-induced stripes is not fixed across the sensor but varies significantly with the distance between the sensor and the object. Furthermore, even when scanning a flat surface, the stripe positions exhibit slight vertical shifts between the top and bottom of the image.

Due to these properties, many existing CFPN reduction techniques—such as guided filtering [20], horizontal differential statistics [21], one-dimensional least squares filtering [22], moment matching [23], and inter-column difference minimization [24]—are not well suited to address the structured, distance-dependent CSN present in profilometer images.

In this article, we propose a method for removing column speckle noise (CSN) from intensity images captured by a profilometer. The method exclusively processes grayscale intensity data, where speckle noise makes it necessary to use a camera in the quality control system in addition to laser profilometers. Our algorithm is specifically designed for flat surfaces, where the speckle noise appears as vertical striping. The approach can also be applied to images of slightly non-planar objects, provided that surface slopes remain moderate, the example is provided at the end of Section 2. However, for objects with complex geometries and steep surfaces, where interference patterns deviate significantly from the vertical direction, the method’s effectiveness is substantially reduced.

The intended application domain includes optical quality control systems, particularly for flat products such as metal sheets or furniture boards transported on conveyor belts. In these contexts, the proposed method enables the use of a single profilometer to simultaneously inspect both geometric features (e.g., surface thickness, bumps, and holes) and visual anomalies (e.g., stains and smudges), within a single scanning pass.

## 2. Materials and Methods

In this section, we present the proposed method for removing column noise from luminance images captured by a profilometer. In the initial version of the algorithm, the correction was performed by subtracting, from each pixel, the difference between the median value of its corresponding column and the global median value of the entire image:

(1) $$a(r,c)=a(r,c)-\underset{i=1,\;\;..,R}{\mathrm{median}}\;a(i,c)+\underset{i=1,\;\;..,R,j=1,\;\;..,C}{\mathrm{median}}\;a(i,j),$$

where *a*(*r*, *c*) is the grayscale level of pixel with coordinates (*r*, *c*), *R* is the number of rows, and *C* is the number of columns of the image.

This correction is conceptually similar to the method proposed in [19], with the key difference being the use of the median instead of the mean. The choice of the median is motivated by the requirements of the target application—namely, the detection of small surface anomalies such as blobs—where preserving sharpness is critical. Using the mean could introduce blurring artifacts, potentially reducing the effectiveness of the visual inspection system.

**Figure 4:**
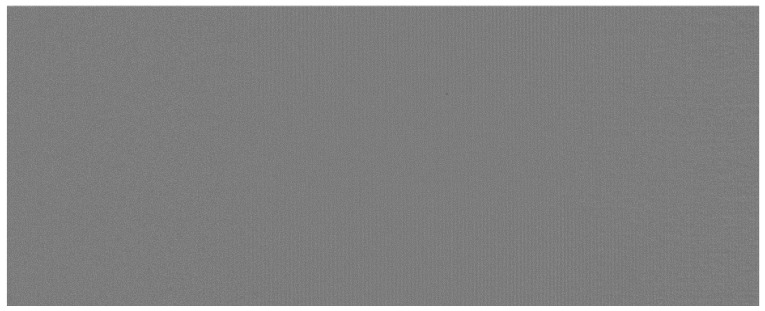
Result of CFPN correction using column median values.

The result of the column noise correction applied to the image from Figure 3 is shown in Figure 4.

It can be observed that some residual noise remains after the initial correction. This is due to the fundamental difference between column speckle noise (CSN) in profilometer grayscale images and column fixed pattern noise (CFPN) typically encountered in line-scan cameras. In images captured with TDI CMOS sensors, CFPN arises from inherent sensor characteristics, resulting in fixed vertical striping patterns that are consistent across all image rows.

In contrast, CSN in profilometer images originates from laser light interference, and the position of the interference-induced stripes varies with the distance between the profilometer and the object surface. Even small variations in this distance can cause the interference pattern to float, resulting in strip positions that gradually shift from the top to the bottom of the image. Consequently, the noise artifacts are not perfectly aligned along vertical columns.

To address this, we refined our algorithm to perform noise compensation locally. In the improved version, the correction is applied based on both column and row indices, allowing for adaptive adjustment across the image:

(2) $$a(r,c)=a(r,c)–\underset{i=R1,\;\;..,R2}{\mathrm{median}}\;a(i,c)+\underset{i=1,\;\;..,R,j=1,\;\;..,C}{\mathrm{median}}\;a(i,j),$$

where *a*(*r*, *c*) is the grayscale level of pixel with coordinates (*r*, *c*), *R* is the number of rows, *C* is the number of columns of the image, *R*1 = max{1, *r-ε*}, *R*2 = min{*R*, *r-ε*}, and *ε* is half of the size of the moving window; for our experiments, we assigned ε = 100.

From an image processing perspective, the output image computed using Equation (2) represents the difference between the original image and its median-filtered version, where the filter uses a vertical mask of size (2ε + 1) × 1. This operation is conceptually related to morphological transformations: analogous operations using minimum and maximum filters are known as white-hat and black-hat transforms, which are commonly used to detect bright or dark features, respectively, that are smaller than the structuring element. These transformations suppress larger features, and similarly, our approach effectively removes grayscale variations that span vertically over a range equal to or greater than (2ε + 1) pixels.

**Figure 5:**
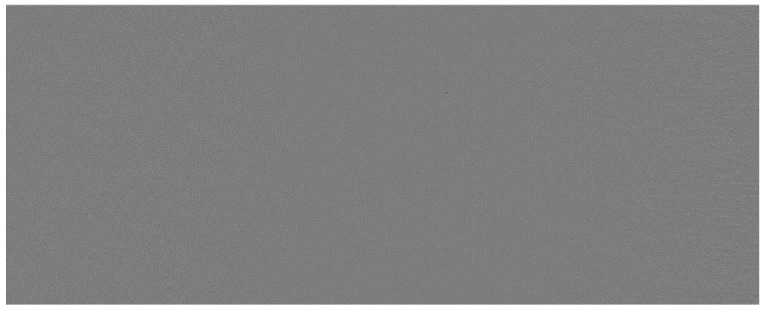
Result of local CFPN correction.

The result of the CSN correction applied to the image from Figure 3 is shown in Figure 5. As can be observed, no residual traces of column fixed pattern noise remain in the corrected image.

**Figure 6:**
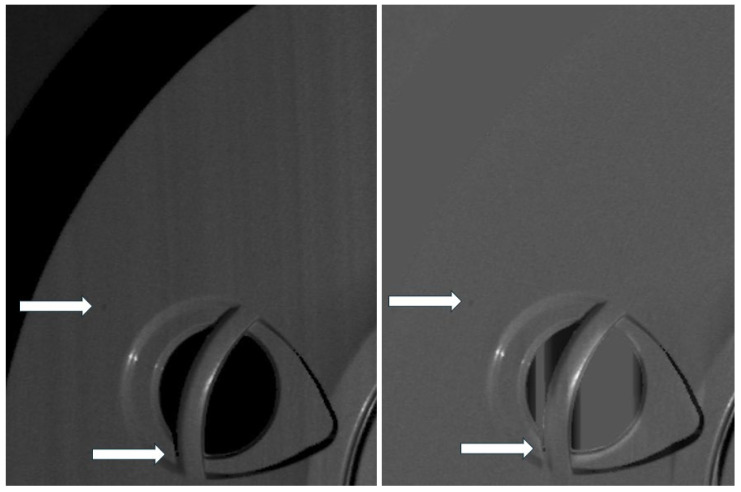
An example of algorithm performance on a 3D object image. White arrows indicate small, isolated features that can be identified as inclusions.

Figure 6 presents an example illustrating the performance of the proposed algorithm on an image of a 3D object containing surface details. Surface inclination does not exceed 15 degrees. The column fixed pattern noise (CFPN) has been effectively removed, along with large-scale luminance variations such as the brightness differences between the object, its shadow, and the background in the top-left region of the image. Importantly, small, isolated features, such as those marked by arrows, are preserved in the output. These features may correspond to surface defects and are critical for detection in optical quality control systems. Particularly, such elements become even more prominent after processing, enhancing the system’s sensitivity to fine anomalies.

A quantitative evaluation of the proposed algorithm is provided in Section 3.

## 3. Results

The performance of the proposed method was evaluated in the context of its target application. To this end, experiments were conducted using flat, homogeneous surfaces scanned with a Keyence LJ-X8400 laser profilometer (Keyence, Osaka, Japan). The evaluation comprises three parts. First, we assess the effectiveness of CSN suppression on flat surfaces using four quantitative metrics. Second, we evaluate the algorithm’s ability to preserve small image details by applying it to a profilometer scan of a flat surface containing artificially introduced blobs of varying sizes. Finally, we demonstrate the integration of the method into a prototype optical quality control system developed for the furniture industry.

### 3.1. The Quality of CFPN Removal for Flat Homogeneous Images

In the following four subsections, we evaluate the effectiveness of column fixed pattern noise (CFPN) removal using five quantitative measures. The first three metrics are specifically designed to assess the reduction of column noise:

- Standard Deviation of the Column Mean Vector (SDCMV), as introduced in [19];
- RMS1, defined by Equation (3) and proposed in this paper;
- RMS2, defined by Equation (4) and also proposed in this paper.

The remaining two metrics are widely used in image quality assessment, particularly for evaluating the fidelity of compressed images relative to a reference:

- Peak signal-to-noise ratio (PSNR);
- Structural Similarity Index Measure (SSIM).

These complementary measures enable a comprehensive analysis of both noise suppression and the preservation of image details.

#### 3.1.1. Standard Deviation of Column Mean Vector

To assess the quality of CSN removal in images of homogeneous surfaces, we used the standard deviation of the column mean vector (SDCMV), as proposed in [19]. This metric quantifies the variation in average intensity across image columns and is particularly effective for evaluating the presence of column noise. The SDCMV values obtained for various objects scanned by the profilometer, using different denoising methods, are summarized in Table 1.

The SDCMV value achieved by our method is 2.5 to 4 times lower than that obtained using the state-of-the-art correction method based on global column mean values, and 2.5 to 5 times lower than when using global column median values. While mean-based correction produces a slightly lower SDCMV compared to median-based correction, it also tends to distort small image elements, features that are critical for flaw detection. In contrast, our approach preserves these fine details while significantly reducing column noise, making it more suitable for high-precision visual inspection tasks.

#### 3.1.2. Fourier Transform

A natural approach to assess the strength and directional characteristics of image interference is through the two-dimensional Fourier transform. For an image of a uniform surface without texture or defects, the Fourier transform should ideally be constant and equal to zero across all frequencies. Non-zero values in the transform indicate irregularities in the image, which, in this context, correspond to noise.

**Figure 7:**
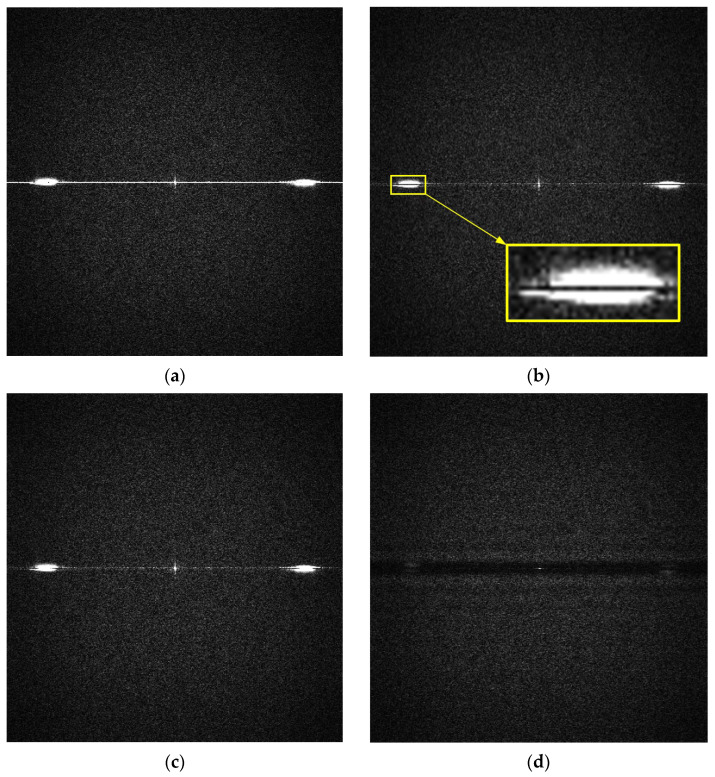
Magnitude of Fourier transform of HDF board image: (**a**) original image taken with profile sensor, (**b**) image after CFPN correction using column mean values, (**c**) image after CFPN correction using column median values, (**d**) image after CFPN correction using local median values.

Figure 7 presents the magnitude spectra of the Fourier transform for the original image of an HDF board and for images denoised using three different methods. In the original image and the images processed using global column mean and column median vector methods, frequency components corresponding to vertical interference fringes are clearly visible. In Figure 7b,c, although horizontal frequencies are attenuated, image elements with even small but non-zero vertical frequency components (F_y_) remain visible, as highlighted in the zoomed region of Figure 7b. The introduction of local compensation in our proposed method enables the removal of column noise that is not perfectly vertical but contains slight vertical frequency components, as demonstrated in Figure 7d.

**Figure 8:**
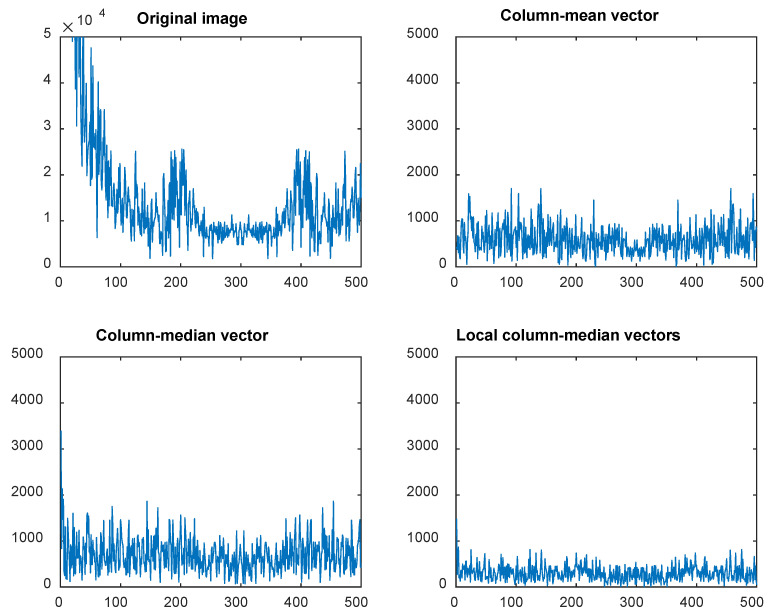
Cross-sections *f* of Fourier spectrum for F_y_ = 0 for different correction methods. Note different scale for original image.

Figure 8 presents cross-sections (*f*) of the Fourier spectrum at F_y_ = 0. It is important to note that the Y-axis scale for the original image is ten times larger than that of the three denoised images.

The root mean square of *f* can be used as a quantitative measure of the noise intensity along the horizontal direction:

(3) $${\mathrm{RMS}}_{1}=\sqrt{\underset{i}{\mathrm{mean}}\left(f_{i}^{2}\right)},$$

Values of RMS_1_ for different methods and various materials are presented in Table 2.

**Figure 9:**
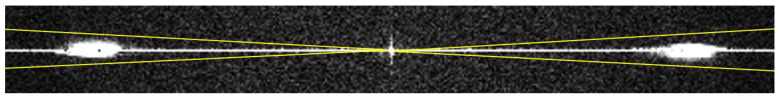
Spectrum of speckle noise. Yellow lines indicate area considered in RMS_2_ coefficient.

RMS_1_ is specifically designed to quantify the effectiveness of removing perfectly vertical strip noise, where the frequency component along the Y- axis is zero. The strength of our method lies in its ability to effectively suppress speckle noise even when the interference strips are slightly tilted. Analysis of sample images acquired with a laser profilometer in the Fourier domain enabled us to define the region in the (F_x_,F_y_) frequency space that corresponds to speckle noise, as illustrated in Figure 9.

Based on this observation, we have developed a measure specifically aimed at assessing the effectiveness of removing column speckle noise from laser profilers:

(4) $${\mathrm{RMS}}_{2}=\sqrt{\underset{x,y:y<0.05x}{\mathrm{mean}}\left(F_{x,y}^{\;2}\right)},$$

Values of RMS_2_ are presented in Table 3.

#### 3.1.3. PSNR and SSIM

The peak signal-to-noise ratio (PSNR) is a widely used metric for assessing image quality after compression or denoising. For an original image A and its noisy approximation B, PSNR is defined as:

(5) $$\mathrm{PSNR}(A,B)=10\;{log}_{10}\left(\frac{max(A)}{MSE(A,B)}\right),$$

where MSE(*A*,*B*) is a mean square error:

(6) $$\mathrm{MSE}(A,B)=\frac{1}{MN}\sum_{i=1}^{M}\sum_{j=1}^{N}{\left(A_{i,j}-B_{i,j}\right)}^{2}.$$

**Figure 10:**
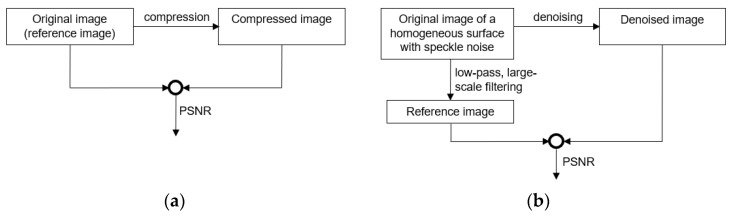
Standard use of PSNR (**a**) and its adaptation to our method (**b**).

PSNR (peak signal-to-noise ratio) cannot be directly applied to our case, as it requires a reference image—typically the original, uncompressed image—to assess the quality of the compressed version based on their similarity (see Figure 10a). However, in our scenario, the original image is already corrupted by speckle noise, and the algorithm aims to remove this noise. Therefore, a true reference image is not available for comparison.

To adapt PSNR for our method, we propose the scheme illustrated in Figure 10b, where a reference image is generated by applying a large-scale low-pass filter to the original image. This filtering process removes the speckle noise, and the resulting output represents the underlying grayscale level of the input image.

This assessment method is valid only for images of homogeneous surfaces, as our algorithm is specifically designed to remove column noise while preserving other image details.

We used 100 × 100 median filter window. Table 4 presents PSNR for different types of surfaces.

Another commonly used metric for assessing image quality is the Structural Similarity Index Measure (SSIM), introduced in [25]. Like PSNR, SSIM is intended to evaluate the difference between an original and a compressed image. Therefore, we adopted the same approach for generating the reference image as shown in Figure 10b.

SSIM produces a value between −1 and 1, where 1 indicates perfect structural similarity, 0 indicates no similarity, and values below 0 suggest negative correlation. The SSIM values obtained in our experiments are presented in Table 5.

#### 3.1.4. Comparison of Measures

Table 6 presents a comparison of metrics averaged across various surface types for three CFPN denoising methods: (a) a state-of-the-art correction using column mean values, (b) correction using column median values, and (c) correction using local median values. The most significant performance improvement is observed in RMS2, a measure specifically designed to quantify this type of noise based on the spectral characteristics of profilometer images. The RMS2 value for the proposed method (c) is over eight times lower than those for methods (a) and (b). SDCMV (as proposed in [19]) and RMS1 are also intended to measure column noise; however, unlike RMS2, they are not tailored to the specific frequency profile of profilometer noise. For the proposed method, RMS1 is reduced by 34% compared to method (a), and SDCMV is nearly three times lower.

The other two metrics, PSNR and SSIM, are general indicators of image quality and are not specifically designed to assess column noise. PSNR, expressed in decibels, increases with similarity and approaches infinity for identical images. The proposed method improves PSNR by 2.12 dB compared to method (a), which is a notable enhancement. SSIM for the proposed method is 0.953, compared to 0.929 for method (a). Since SSIM values range from −1 to 1, with 1 indicating perfect similarity, it is more meaningful to consider how closely the result approaches 1 rather than comparing ratios. From this perspective, the improvement is substantial.

**Figure 11:**
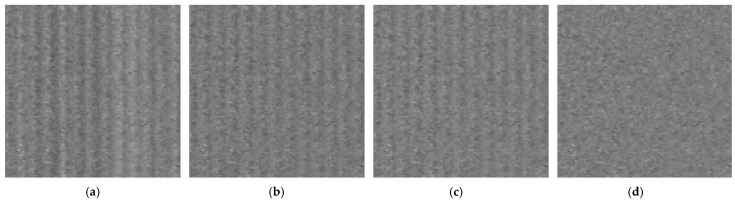
Visual comparison of methods: (**a**) original image, (**b**) CFPN correction using column mean values, (**c**) CFPN correction using column median values, (**d**) CFPN correction using local median values.

Visual comparison of the results is presented in Figure 11.

#### 3.1.5. Sensitivity to Parameter ε

**Figure 12:**
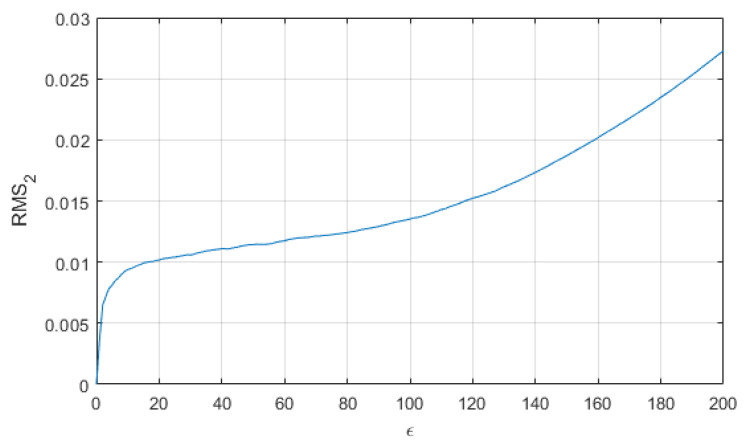
Sensitivity of measure RMS_2_ to parameter ε.

In this chapter, we examine how the performance of our algorithm varies with the parameter ε, which represents half of the window size and defines *R1* and *R2* in Equation (2). The analysis uses the RMS_2_ measure, specifically designed to assess the effectiveness of removing column speckle noise from laser profiler images. The results are shown in Figure 12.

Noise removal is most effective at small values of ε. When ε = 0, the output image becomes homogeneous, with all pixel values equal to the median of the input image, resulting in an RMS_2_ value of zero. Although this completely eliminates column noise, it also removes all image details. The RMS_2_ value increases monotonically as ε grows. The increase is rapid between 0 and 15, then slows considerably between 20 and 100, after which it steepens again for larger ε values.

The choice of ε involves a trade-off between the effectiveness of speckle noise removal and preserving potentially relevant image details for subsequent analysis. Details with a horizontal span smaller than ε will be preserved, while larger features may be removed according to Equation (2). We set ε = 100 to ensure that all irregularities in the image, which could represent potential flaws, are accurately detected.

### 3.2. Preserving Relevant Details of the Image

**Figure 13:**
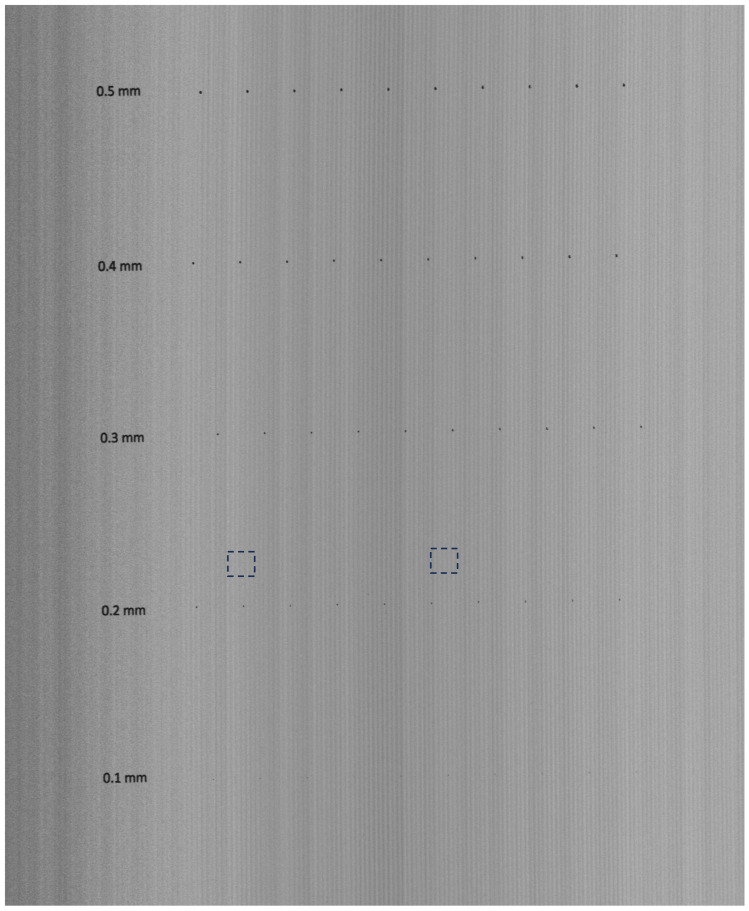
Image of test surface from a Keyence LJ-X8400 laser profilometer. Areas marked with dashed rectangles are shown in Figure 14, zoomed in.

To verify the ability of the method to preserve the details of the image, we prepared a test image printed on an aluminum board (Dibond), see Figure 13. The test image consists of circles with diameters of 0.5, 0.4, 0.3, 0.2, and 0.1 mm.

**Figure 14:**
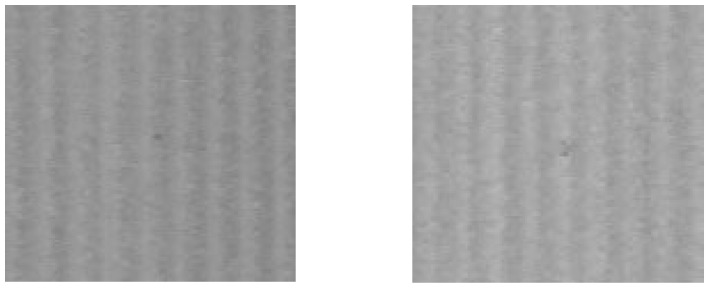
Zooming of two selected areas from Figure 13 with a 0.1 mm dot in the middle.

To illustrate the size of the dots relative to the interference strips, two areas marked with dashed squares in Figure 13 are shown in Figure 14. Each area contains a 0.1 mm dot at the center, which should remain unaffected by the speckle noise removal algorithm and subsequently be automatically detected.

**Figure 15:**
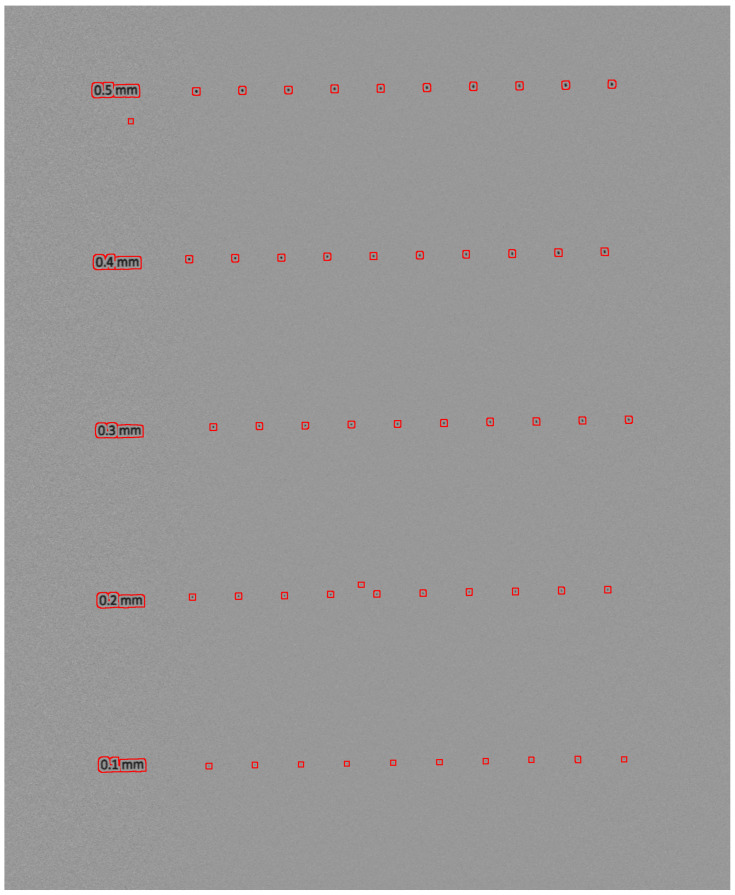
Automatic detection of surface flaws in image from Figure 13 after removing interference fringes.

Figure 13 served as the input for our algorithm. After removing the interference fringes, the processed image was used as input for an optical surface defect detection system. Defects appear as small regions with luminance differing from the surrounding surface. The flaw detection algorithm relies on a series of simple morphological operations, which we do not describe here as they fall outside the scope of this paper. The results are shown in Figure 15. The interference fringes were successfully removed, while details such as small dots—down to 0.1 mm in size—remained unaffected and were all correctly detected.

Original images and the results of the experiments presented in this chapter are available as graphic files in the Supplementary Materials.

### 3.3. Application of the Proposed Method for the Detection of Blobs in the Optical Inspection System

We applied the proposed method in a prototype optical quality control system for the furniture industry, where the inspected items include laminated fiberboards and other furniture components. The optical inspection covers both the geometry of the boards and any surface irregularities reflected in grayscale variations. The system employs a Keyence LJ-X8400 laser profilometer to monitor the geometry of each inspected element. Our goal was also to use this device to detect visual surface flaws, identified as local changes in grayscale intensity.

**Figure 16:**
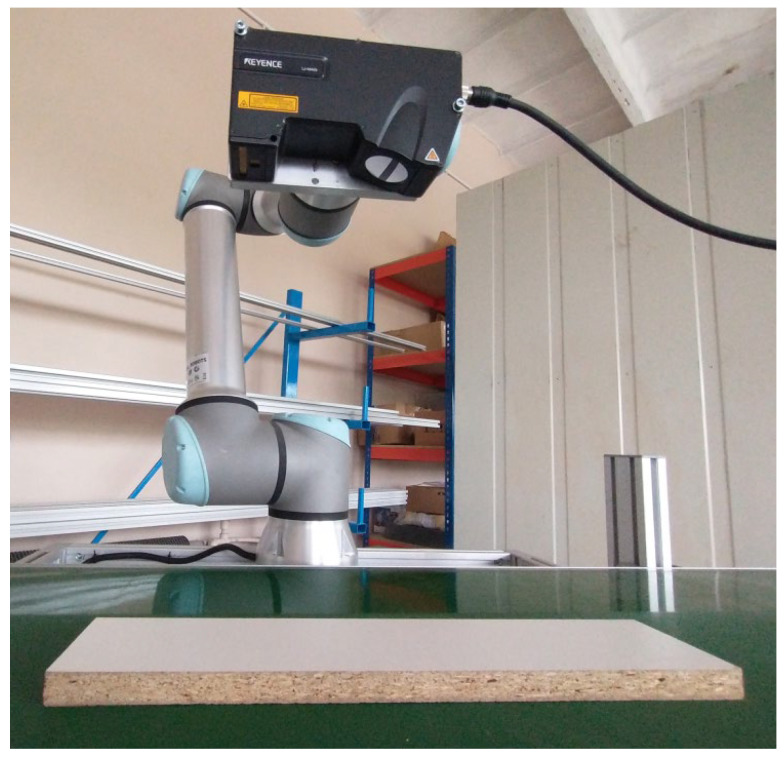
Laboratory stand for simultaneous control of geometrical and visual defects of flat surfaces. A fiberboard is prepared on the conveyor belt for scanning.

The elements move along a conveyor belt, with their position tracked by an incremental encoder providing a resolution of 2.1 μm. The profilometer is mounted on a robotic arm to enhance system flexibility, allowing adjustment of the trade-off between field of view and measurement accuracy during operation. Figure 16 shows the laboratory setup with a fiberboard ready for scanning.

**Figure 17:**
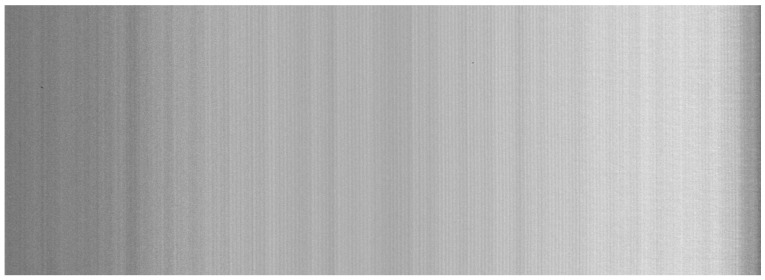
A photo of fiberboard taken by profilometer, affected by speckle noise.

Along with the 3D scan in the form of a point cloud, the profilometer also captured an image of the board. This image is significantly affected by column speckle noise, as illustrated in Figure 17.

**Figure 18:**
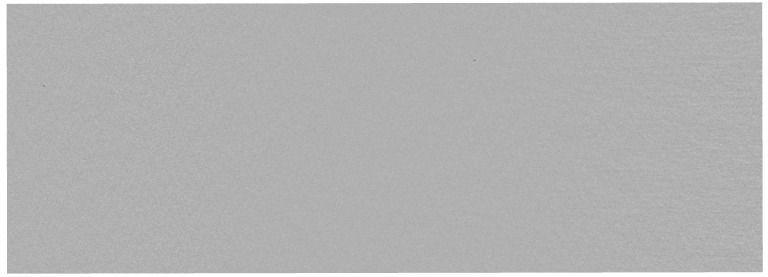
Result of our denosing algorithm for photo in Figure 17.

**Figure 19:**
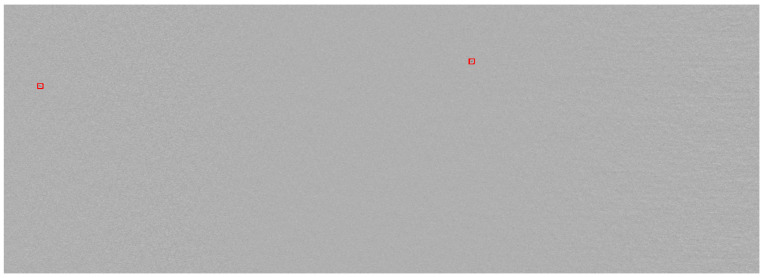
Visual flaws detected in fiberboard photo taken by profilometer.

The image shown in Figure 18 was used as input for the system designed for optical detection of surface defects, which appear as small areas with luminance differing from the surrounding surface. The flaw detection algorithm is based on a series of simple morphological operations; however, its detailed description is not included here, as it falls outside the scope of this paper. Figure 19 presents the result of the automatic detection of surface flaws in the board image from Figure 13, following the application of the proposed method for removing interference fringes.

The algorithm’s processing time is low enough to be integrated into a quality control system without negatively affecting overall throughput. Without parallel computing, the processing time is proportional to the number of pixels in the input image. The largest images captured by the profilometer had a resolution of 3018 × 2353 pixels, and the processing time for such images on a standard PC was 0.37 s. This allows the algorithm to operate in real time with a significant margin, given a scanning speed of 20 cm/s and a total scan duration of 2 s per element. Since the primary computational load arises from a form of median filtering, the algorithm is well-suited for parallelization. In scenarios requiring faster processing, it can be implemented on GPU or FPGA hardware.

## 4. Discussion

In this article, we proposed a method for removing column speckle noise (CSN) from 2D images acquired by laser profilometers. These images are captured simultaneously with the primary data—three-dimensional point clouds—during a single scan. However, they are often affected by strong column noise caused by optical interference. Unlike column noise in CMOS TDI cameras, where vertical strip positions are fixed, in profilometer images the strip positions vary depending on the distance to the scanned surface. Consequently, no standard method currently exists for effectively removing speckle noise in profilometer 2D imagery, and manufacturers typically do not offer any built-in tools to improve image quality. As a result, the 2D imaging capability of profilometers is rarely used, and system designers often choose to add separate cameras for visual inspection.

Our target application involves flat surfaces, where horizontal variation of speckle noise is minimal. This allows for column-wise compensation. However, even minor positional shifts of the interference patterns make it impractical to directly apply conventional column fixed pattern noise (CFPN) correction methods developed for TDI CMOS sensors.

To address this, we proposed a localized speckle noise reduction algorithm. It compensates for the interference by subtracting from each pixel the median grayscale value of its column, computed within a local vertical window around the pixel. This is further adjusted by the global median of the entire image.

The method is specifically tailored for flat, homogeneous surfaces, although it can also be applied to 3D objects with limited depth variation. In addition to removing speckle noise, the algorithm effectively suppresses large-scale grayscale gradients, producing a differential-like output image. Therefore, it should not be considered a general-purpose denoising tool. However, small-scale variations—such as those indicating surface flaws—are preserved, making the method highly suitable for optical quality control tasks.

The primary application is in the furniture industry, where the method has been successfully integrated into a fiberboard flaw detection system. Importantly, the same laser profilometer is used simultaneously for both 3D and 2D inspection tasks.

Future developments could include adapting the proposed method for denoising images of 3D objects with steep or complex surfaces. In such cases, the direction of interference strips is no longer strictly vertical but varies depending on surface geometry. To address this, the algorithm could be extended to follow the direction of the strips dynamically, adjusting the orientation of the moving window accordingly.

Another promising direction for future work is the development of an alternative denoising approach based on deep neural networks. A major challenge here is the lack of suitable training data: while profilometer-acquired images affected by column speckle noise can serve as input, corresponding clean ground truth images are typically unavailable. One potential solution is to use high-quality images taken with standard cameras as clean references and synthetically add column speckle noise to create paired training samples. Alternatively, unsupervised learning techniques could be explored, leveraging the known spectral characteristics of CSN to guide the training process without requiring explicit ground truth.

## Figures and Tables

**Table 1:** Standard deviation of column mean vector (SDCMV) for different surfaces and different correction methods.

	Original Image	CFPN Correction Using Column Mean Values	CFPN Correction Using Column Median Values	CFPN Correction Using Local Median Values
White furniture board	18.7381	0.2893	0.3198	0.1095
HDF board	20.7926	0.29292	0.53735	0.10033
Painted fiberboard	17.3543	0.29175	0.32734	0.12376
Aluminum board	4.7499	0.29174	0.37502	0.07498
White plastic sheet	19.9029	0.28436	0.41664	0.09236

**Table 2:** RMS given by (3) for different surfaces and different correction methods.

	Original Image	CFPN Correction Using Column Mean Values	CFPN Correction Using Column Median Values	CFPN Correction Using Local Median Values
White furniture board	110,126	688	836	383
HDF board	284,145	1729	4698	1024
Painted fiberboard	278,427	1826	2576	1560
Aluminum board	31,462	757	1288	335
White plastic sheet	213,800	1219	2385	761

**Table 3:** RMS_2_ given by Equation (4) for different surfaces and different correction methods.

RMS_2_	Original Image	CFPN Correction Using Column Mean Values	CFPN Correction Using Column Median Values	CFPN Correction Using Local Median Values
White furniture board	0.422649	0.191576	0.191856	0.033532
HDF board	1.403035	0.521778	0.538224	0.056508
Painted fiberboard	1.473558	0.186979	0.190485	0.047434
Aluminum board	0.412809	0.215616	0.217044	0.013529
White plastic sheet	0.954164	0.346692	0.350378	0.030150

**Table 4:** PSNR [dB] for different surfaces and different correction methods.

	Original Image	CFPN Correction Using Column Mean Values	CFPN Correction Using Column Median Values	CFPN Correction Using Local Median Values
White furniture board	38.1398	42.1674	42.1614	44.0632
HDF board	35.9574	39.1019	39.0538	41.6184
Painted fiberboard	36.4712	38.5002	38.6035	39.6811
Aluminum board	38.3319	43.0832	43.3415	46.5082
White plastic sheet	35.0886	40.8529	40.8324	42.9152

**Table 5:** SSIM for different surfaces and different correction methods.

	Original Image	CFPN Correction Using Column Mean Values	CFPN Correction Using Column Median Values	CFPN Correction Using Local Median Values
White furniture board	0.90963	0.94074	0.94072	0.96129
HDF board	0.85332	0.88788	0.88859	0.93502
Painted fiberboard	0.87552	0.93619	0.93614	0.94076
Aluminum board	0.89927	0.9542	0.95408	0.97734
White plastic sheet	0.85845	0.92628	0.92614	0.95132

**Table 6:** Comparison of measures averaged over all types of surface.

	Original Image	(a) CFPN Correction Using Column Mean Values	(b) CFPN Correction Using Column Median Values	(c) CFPN Correction Using Local Median Values
SDCMV	16.30756	0.290014	0.39523	0.100186
RMS_1_	183,592	1243.8	2356.6	812.6
RMS_2_	0.933243	0.292528	0.297597	0.036231
PSNR	36.79778	40.74112	40.79852	42.95722
SSIM	0.879238	0.929058	0.929134	0.953146

## Data Availability

Data are contained within the article and Supplementary Materials.

## Supplementary Materials

The following supporting information can be downloaded at: https://www.mdpi.com/article/10.3390/s25113426/s1, Original images from laser profilometers and the output images from noise correction algorithms compared in the article, for various materials.
