# Supplementary figures and images for: Compensation of Speckle Noise in 2D Images from Triangulation Laser Profile Sensors Using Local Column Median Vectors with an Application in a Quality Control System
Source: Sensors (Basel). 2025 May 29;25(11):3426. doi: 10.3390/s25113426 (PMC12158334; doi:10.3390/s25113426)

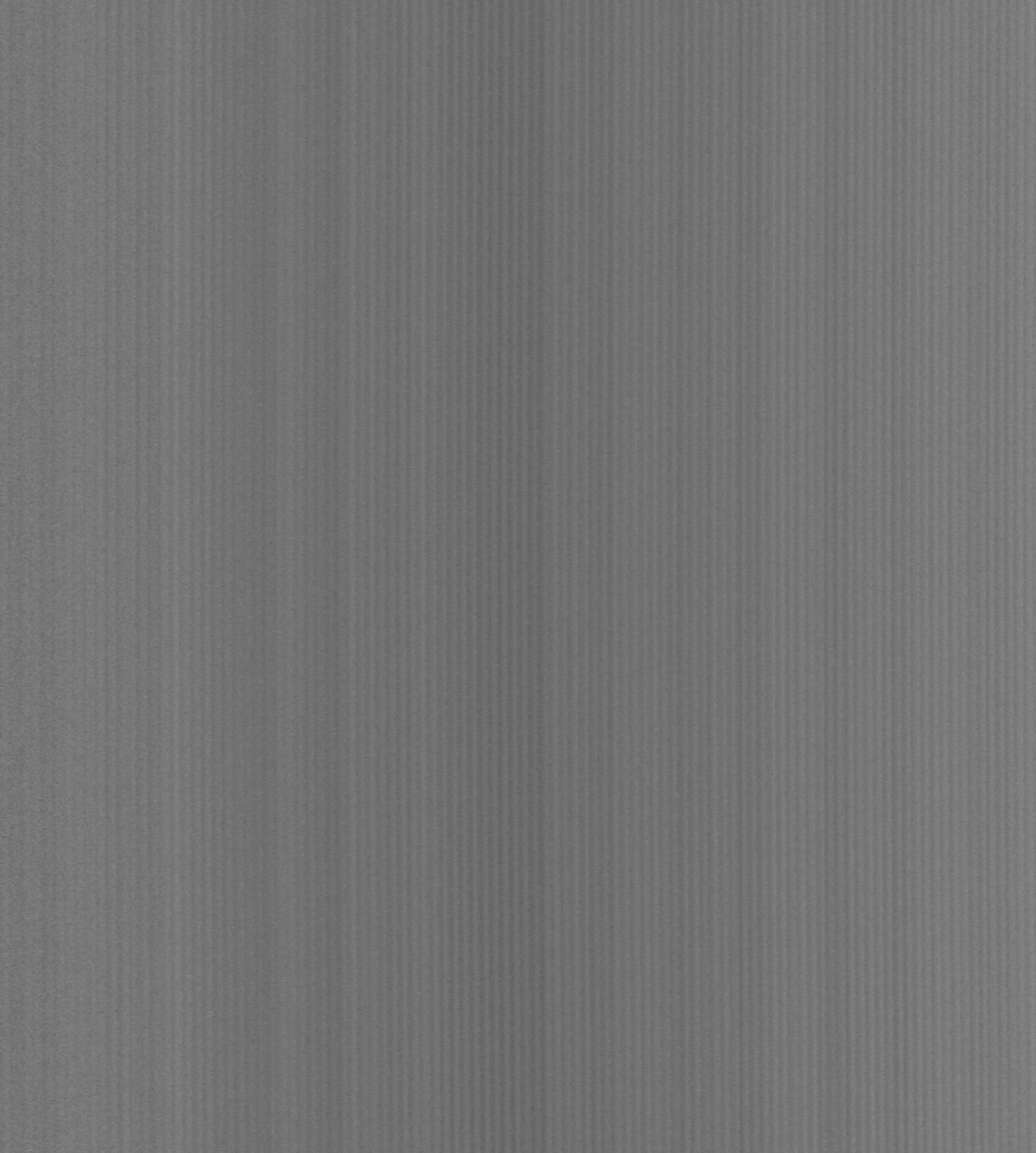

Supplement: Supplementary file 1 [file sensors-25-03426-s001.zip › AluminiumBoard/AluminiumBoard_0_original.tif]

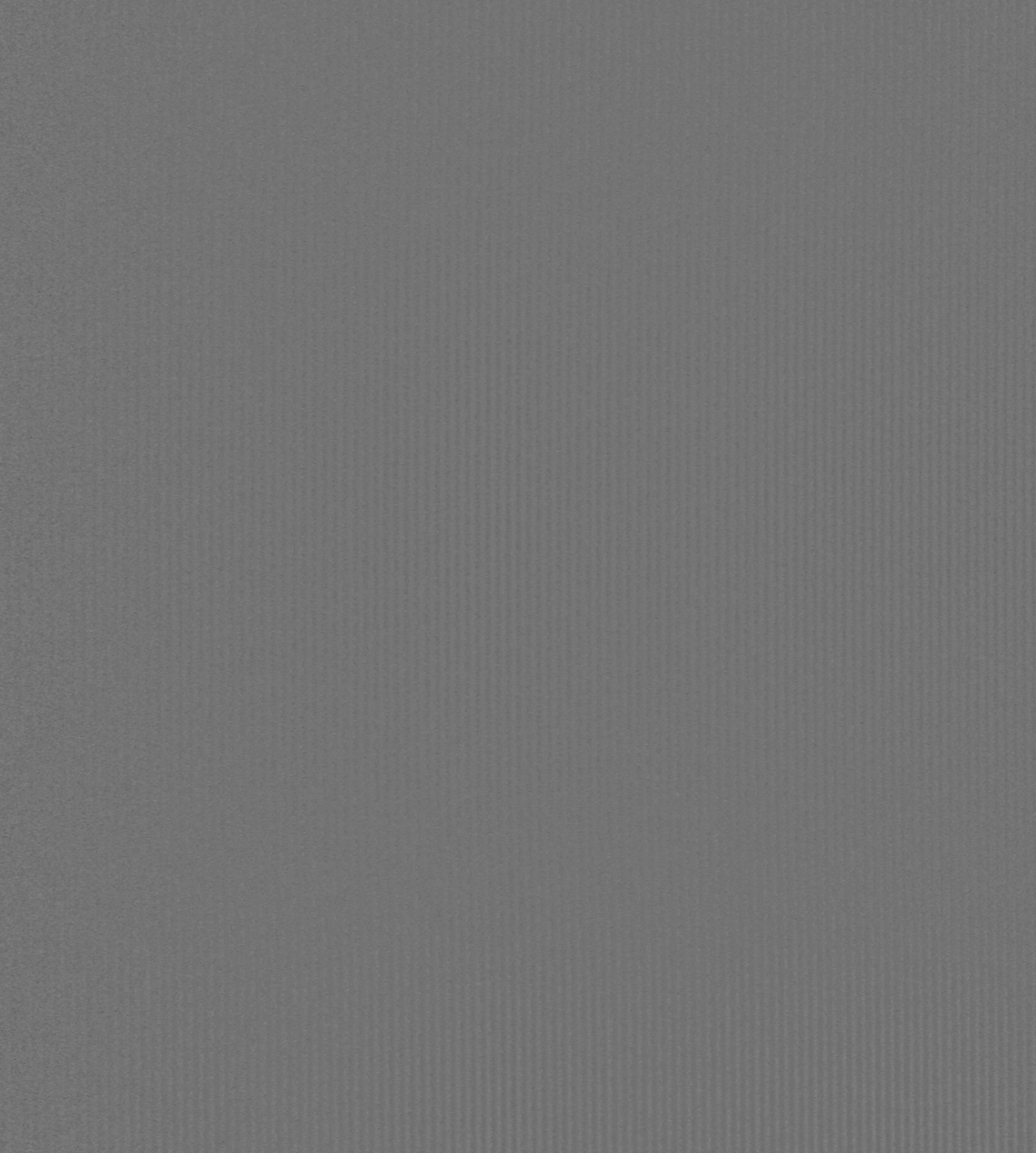

Supplement: Supplementary file 1 [file sensors-25-03426-s001.zip › AluminiumBoard/AluminiumBoard_1_mean.tif]

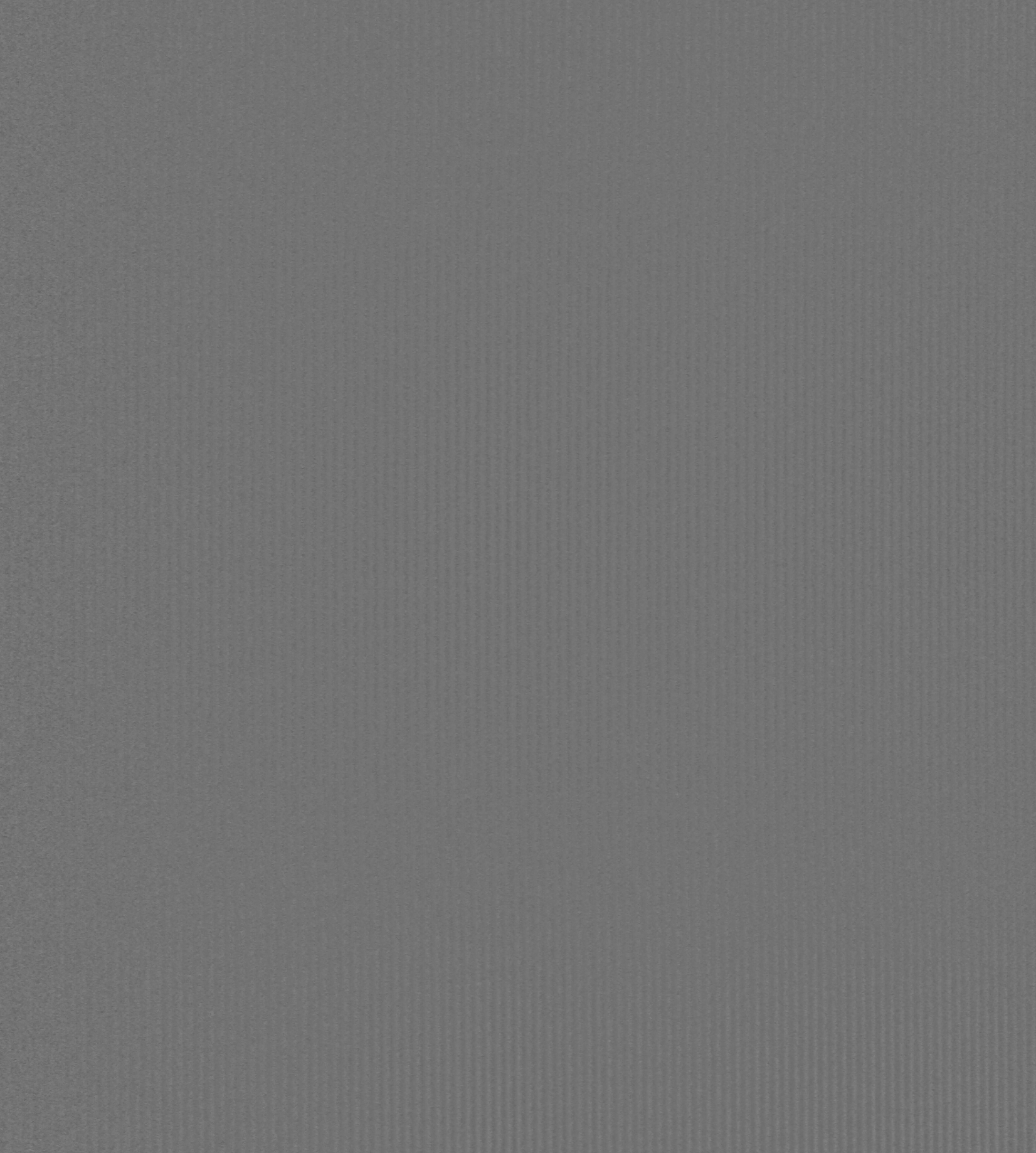

Supplement: Supplementary file 1 [file sensors-25-03426-s001.zip › AluminiumBoard/AluminiumBoard_2_median.tif]

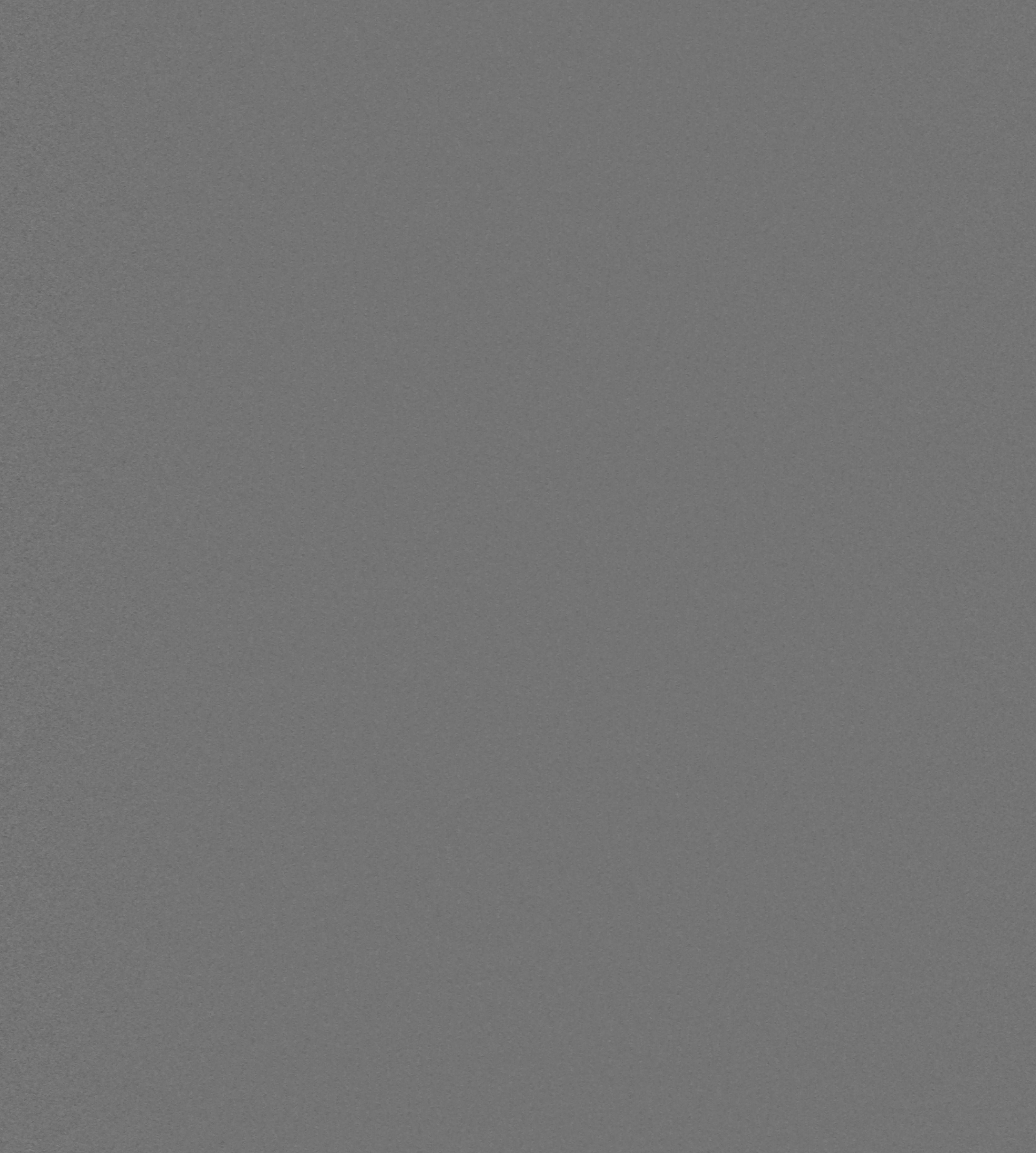

Supplement: Supplementary file 1 [file sensors-25-03426-s001.zip › AluminiumBoard/AluminiumBoard_3_local_median.tif]

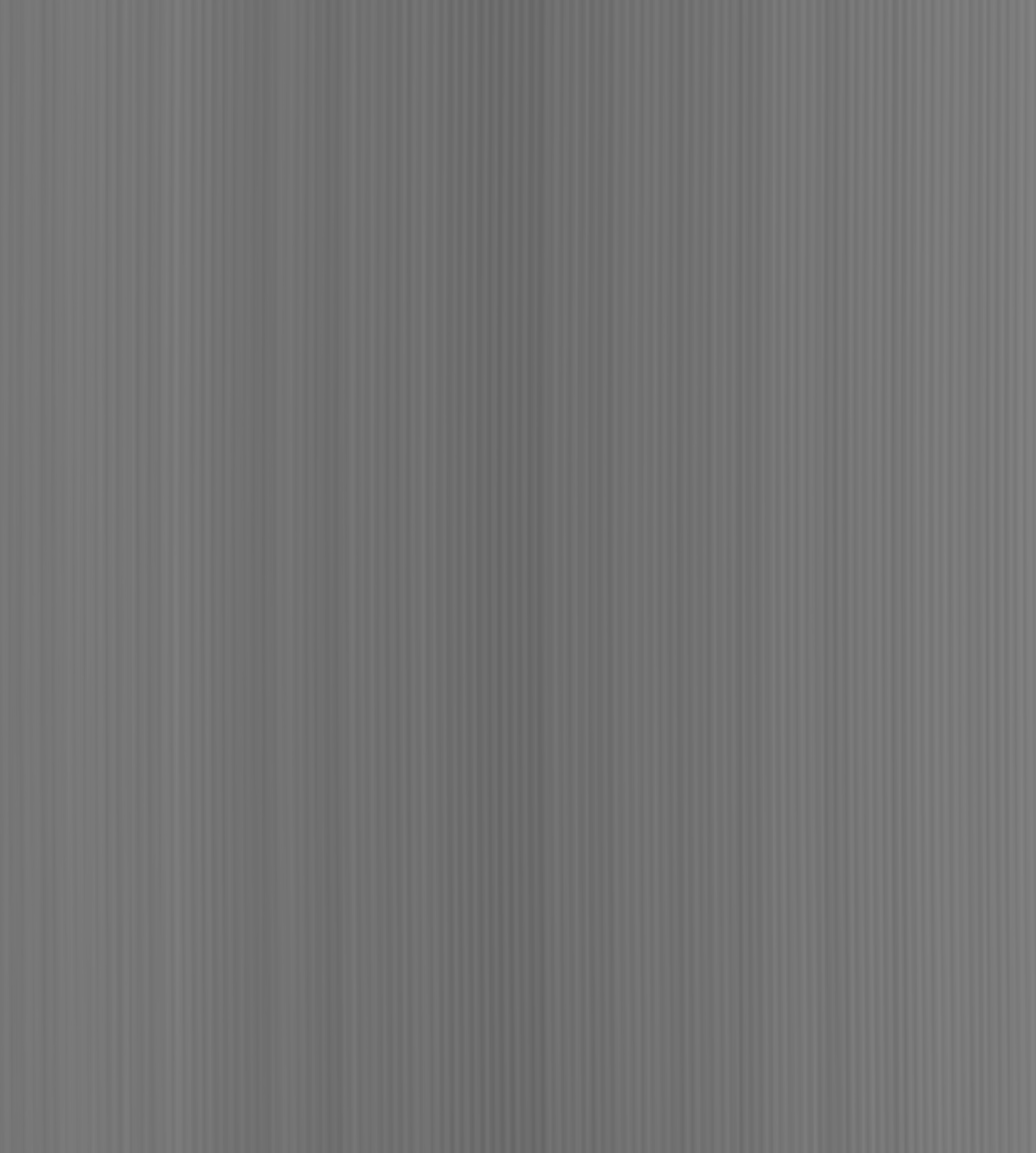

Supplement: Supplementary file 1 [file sensors-25-03426-s001.zip › AluminiumBoard/AluminiumBoard_4_median_image.tif]

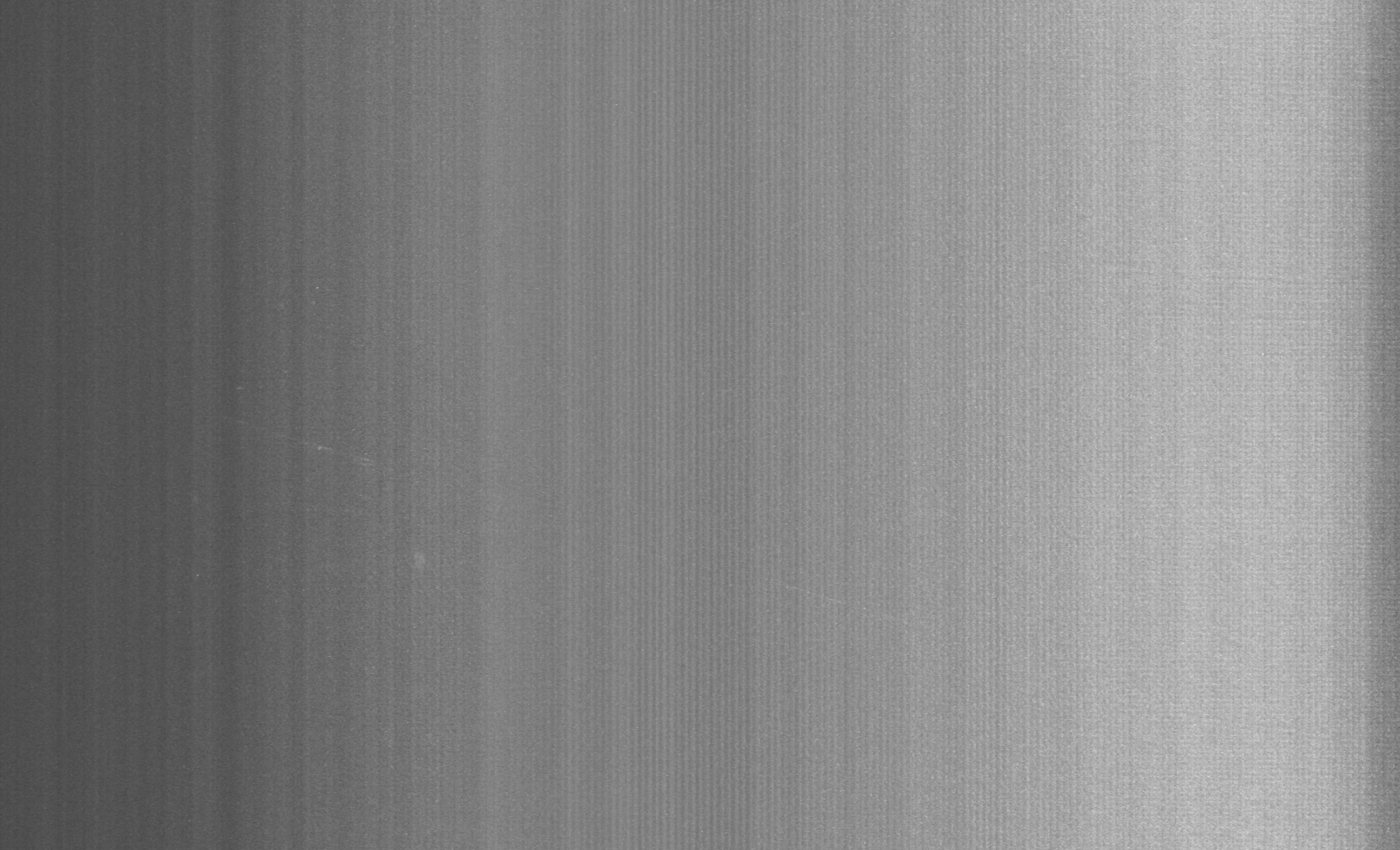

Supplement: Supplementary file 1 [file sensors-25-03426-s001.zip › ConveyorBelt/ConveyorBelt_0_original.tif]

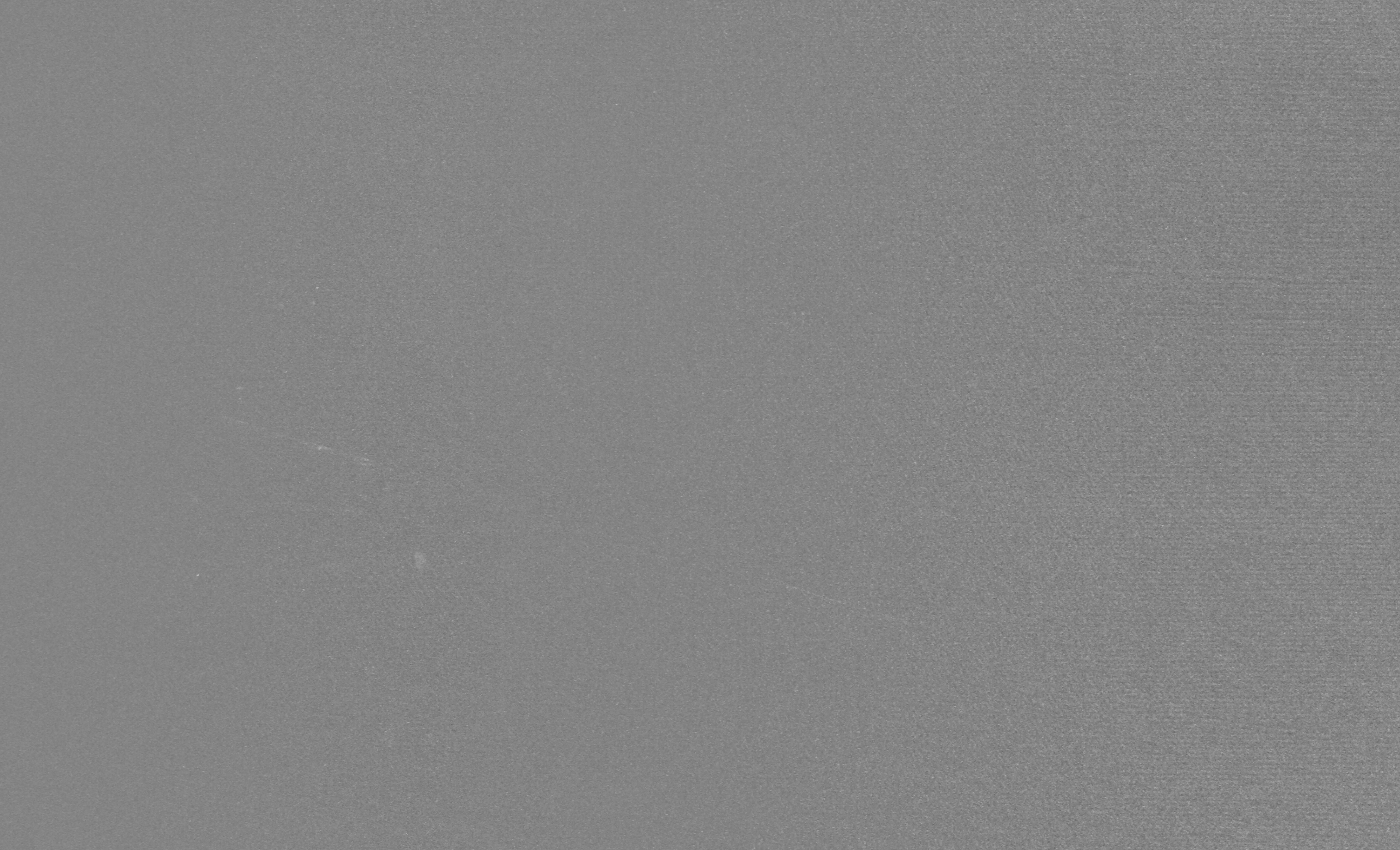

Supplement: Supplementary file 1 [file sensors-25-03426-s001.zip › ConveyorBelt/ConveyorBelt_1_mean.tif]

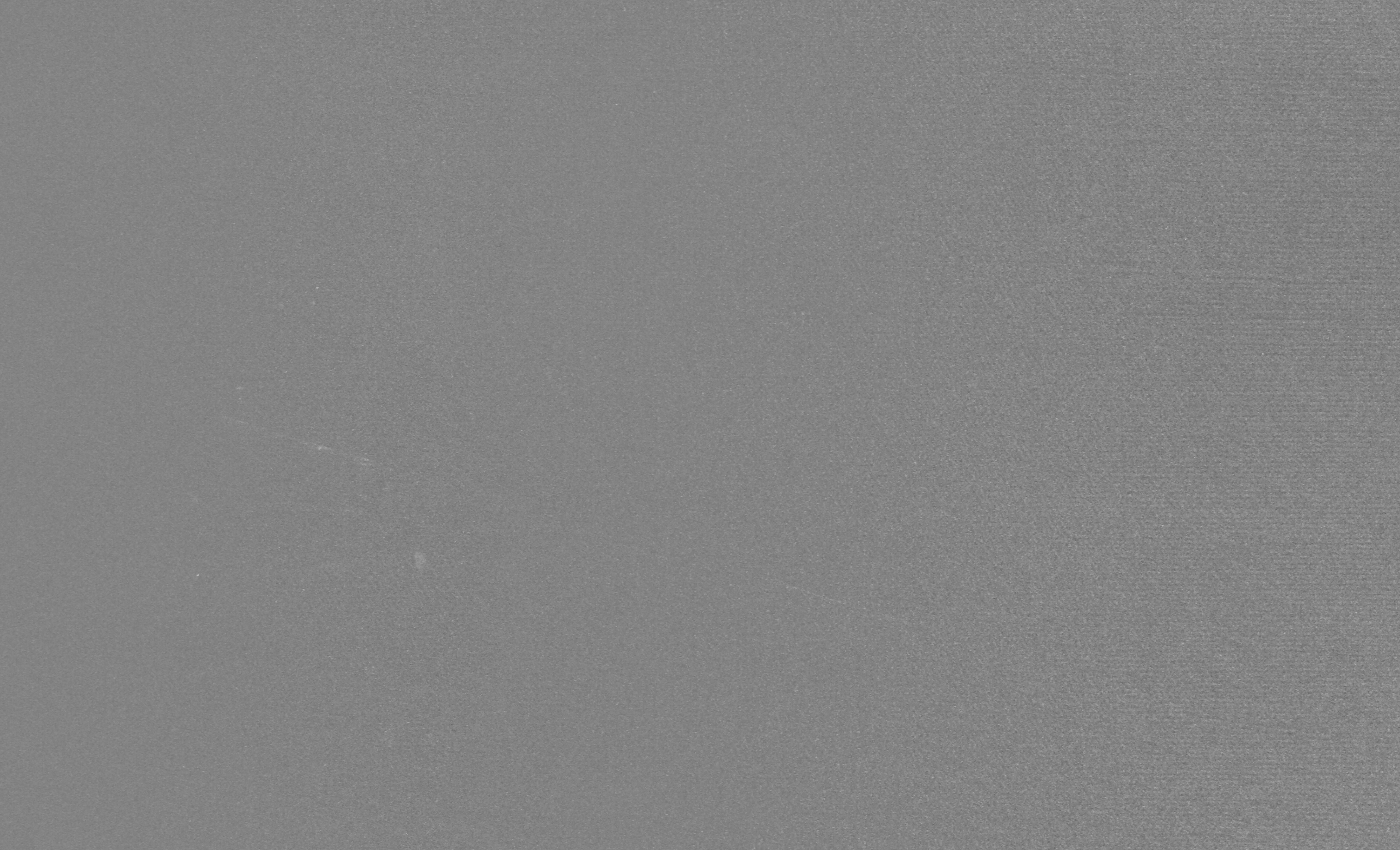

Supplement: Supplementary file 1 [file sensors-25-03426-s001.zip › ConveyorBelt/ConveyorBelt_2_median.tif]

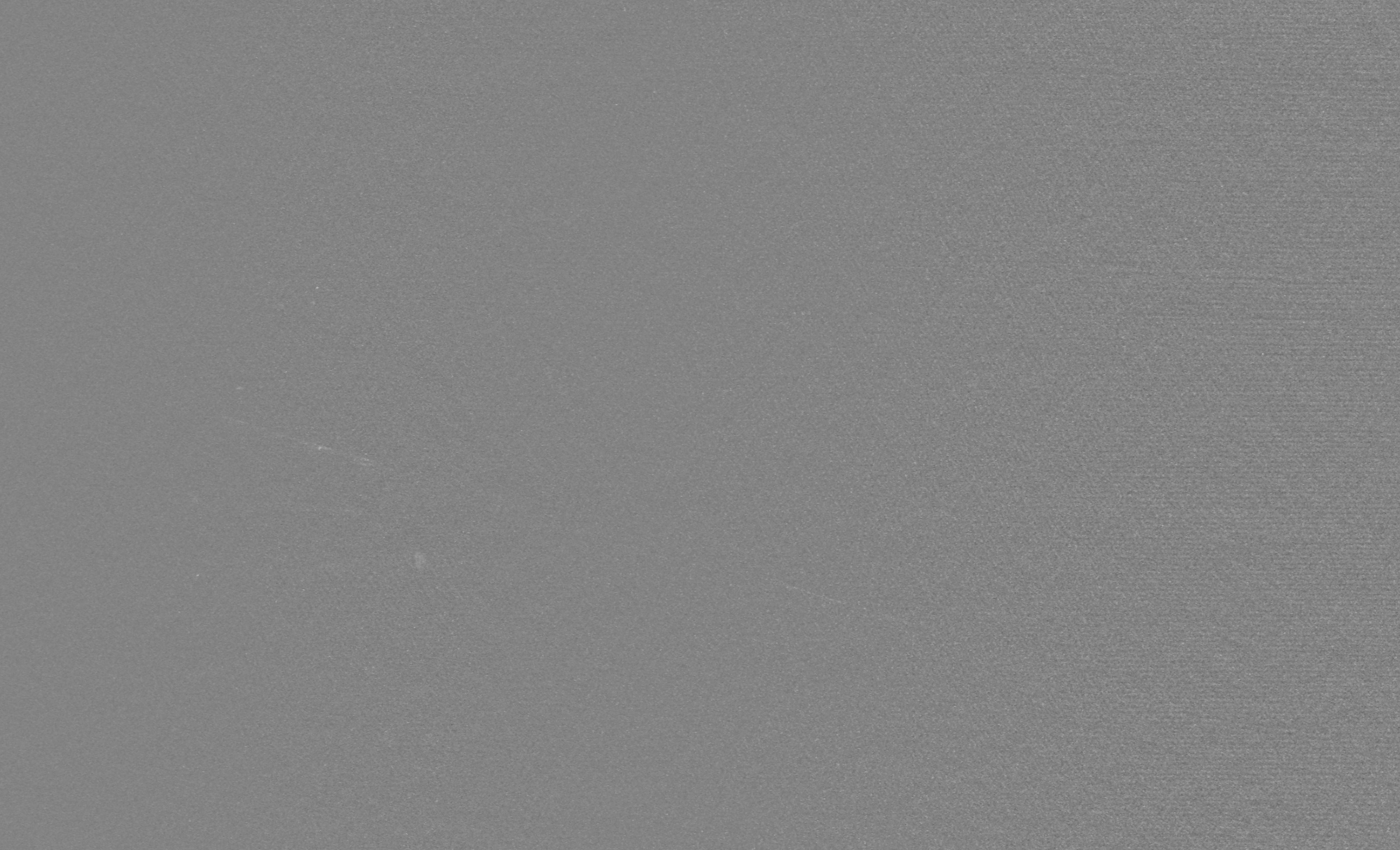

Supplement: Supplementary file 1 [file sensors-25-03426-s001.zip › ConveyorBelt/ConveyorBelt_3_local_median.tif]

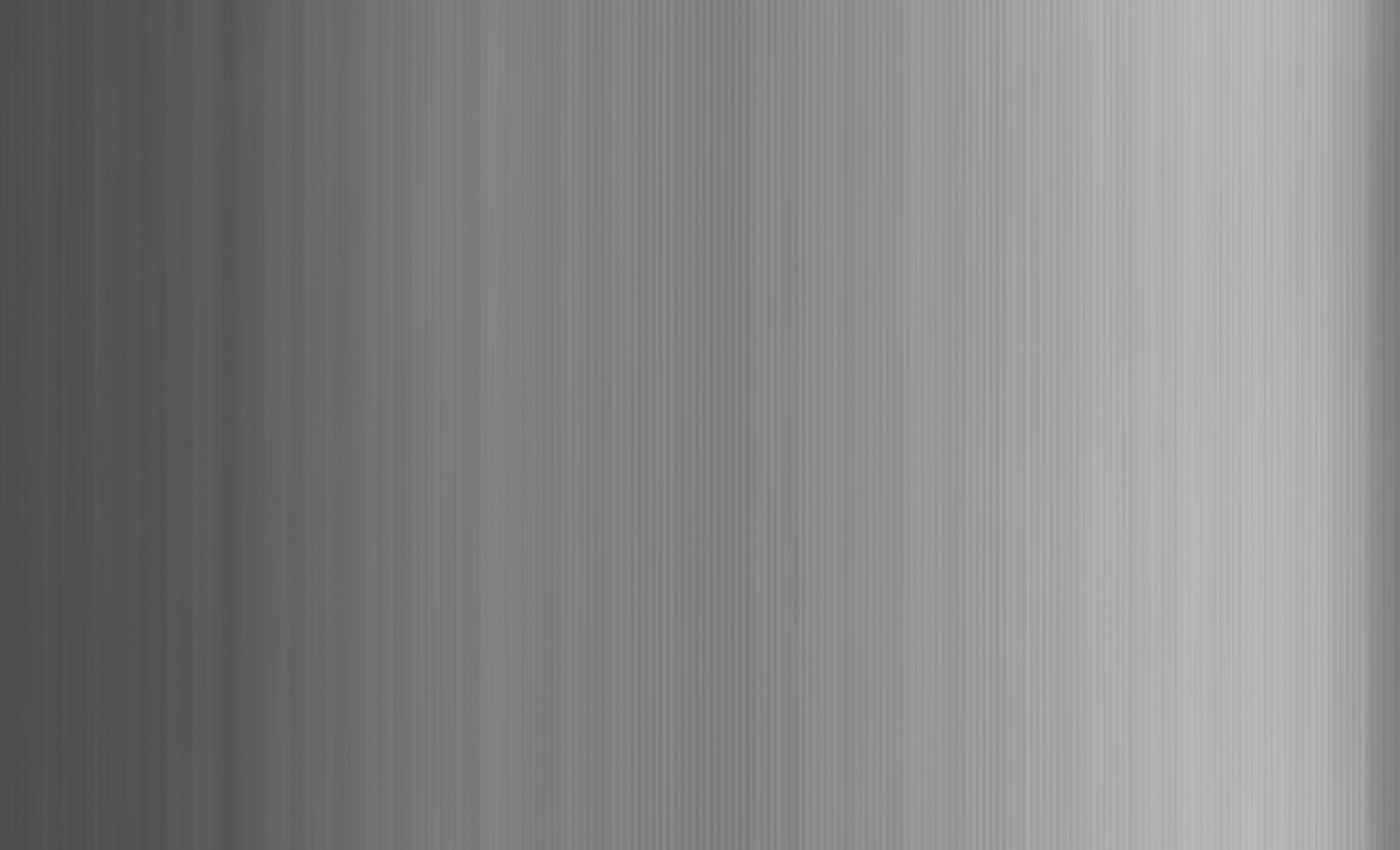

Supplement: Supplementary file 1 [file sensors-25-03426-s001.zip › ConveyorBelt/ConveyorBelt_4_median_image.tif]

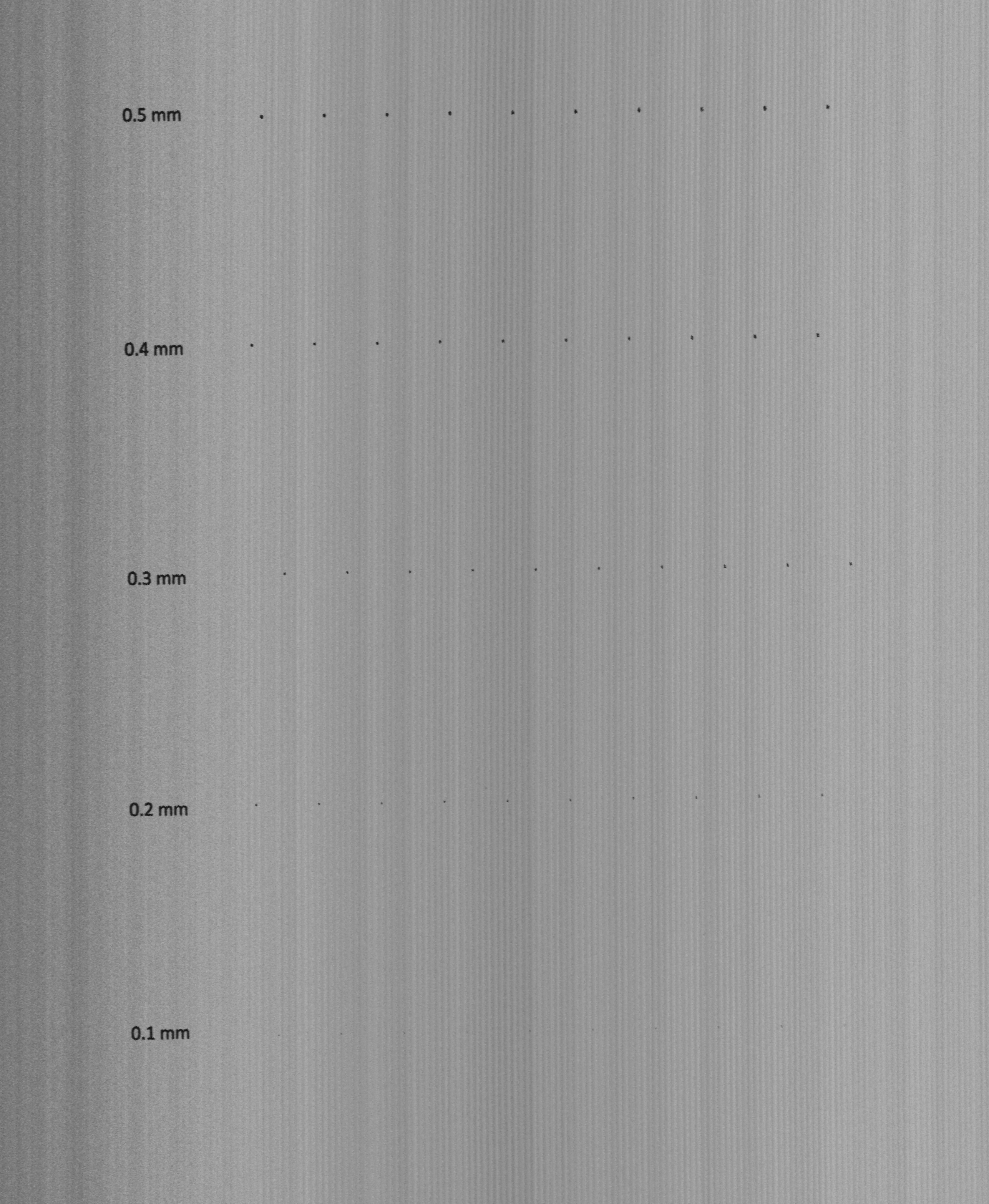

Supplement: Supplementary file 1 [file sensors-25-03426-s001.zip › DotsOnDibond/DotsOnDibond_0_original.tif]

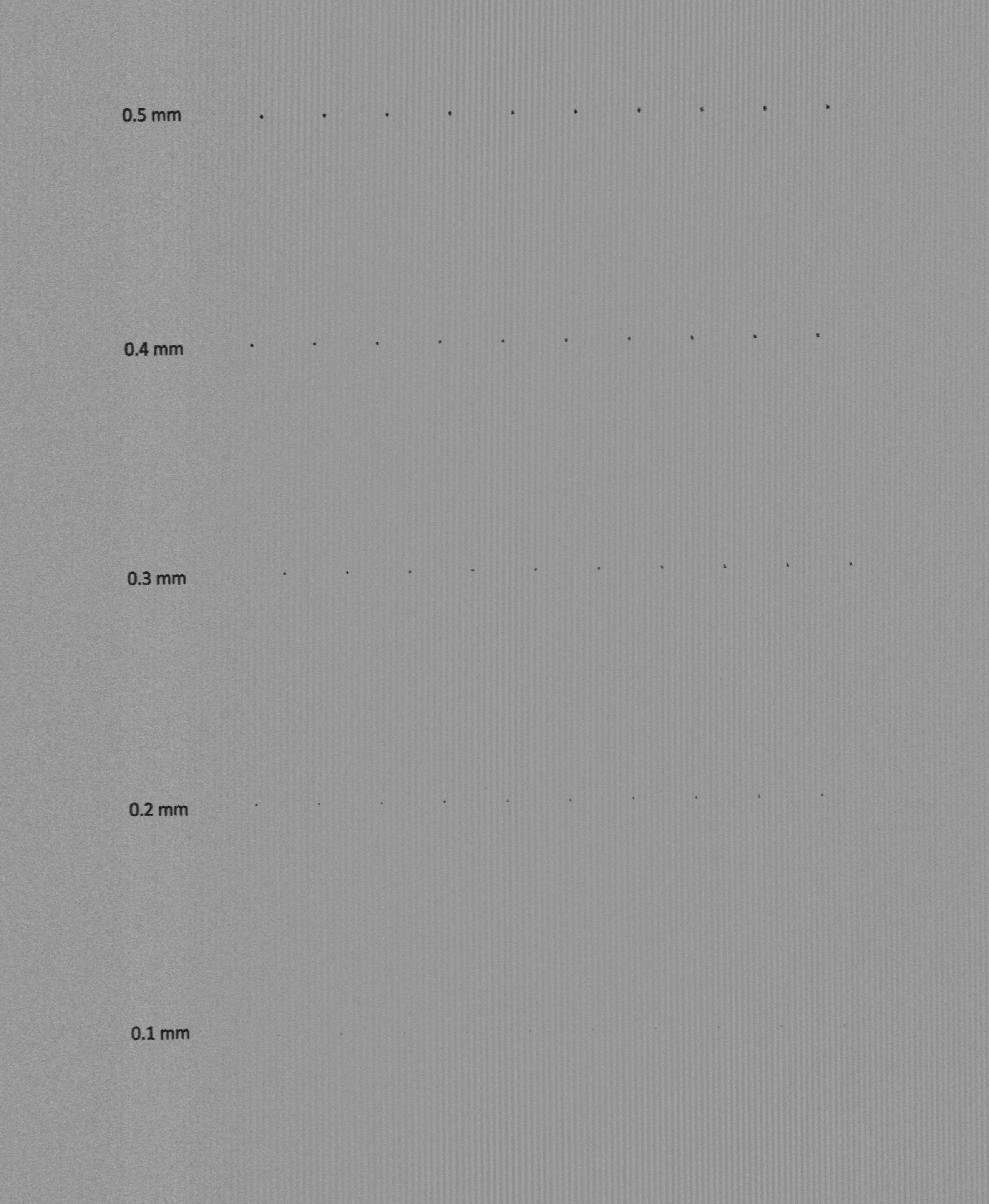

Supplement: Supplementary file 1 [file sensors-25-03426-s001.zip › DotsOnDibond/DotsOnDibond_1_mean.tif]

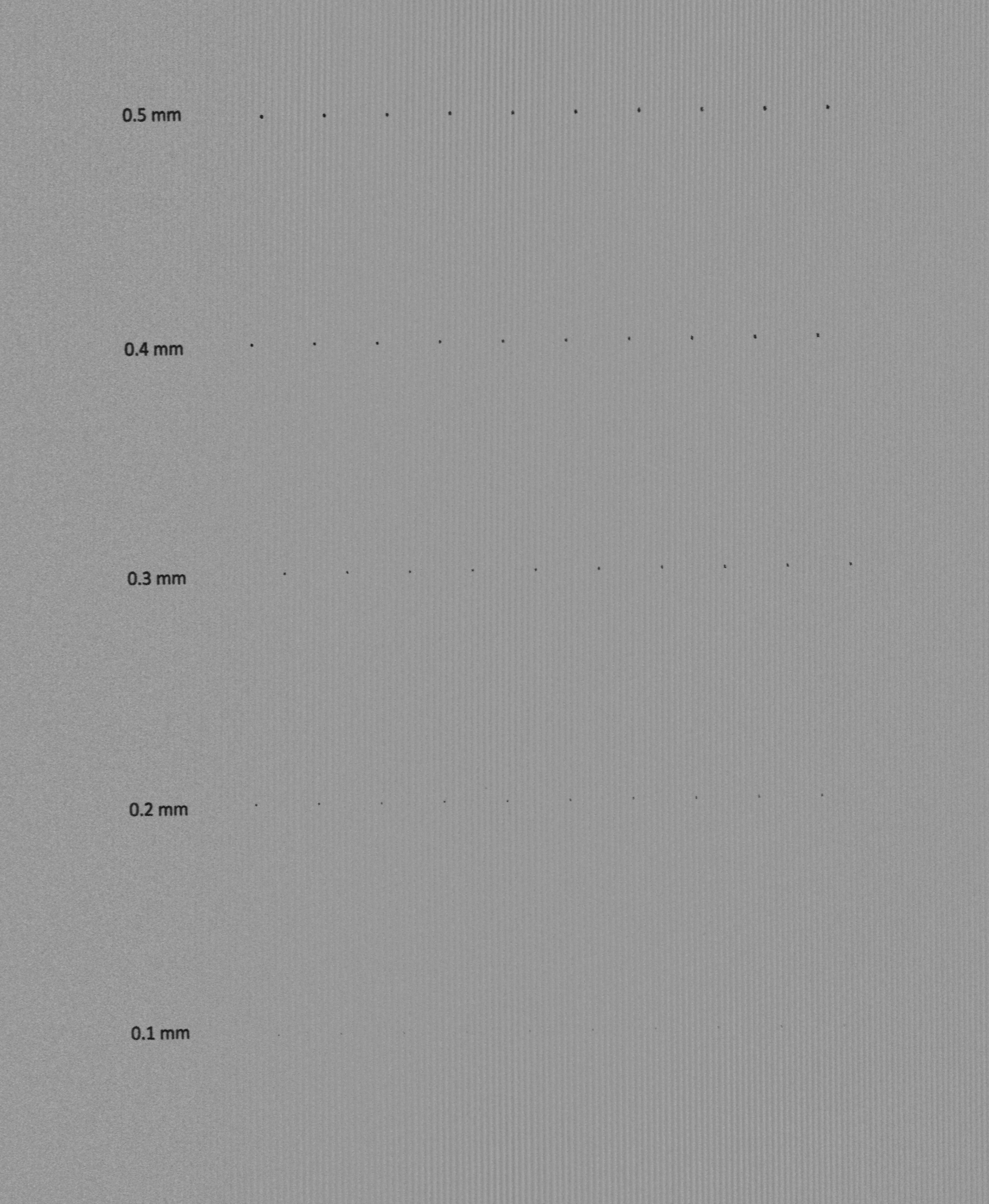

Supplement: Supplementary file 1 [file sensors-25-03426-s001.zip › DotsOnDibond/DotsOnDibond_2_median.tif]

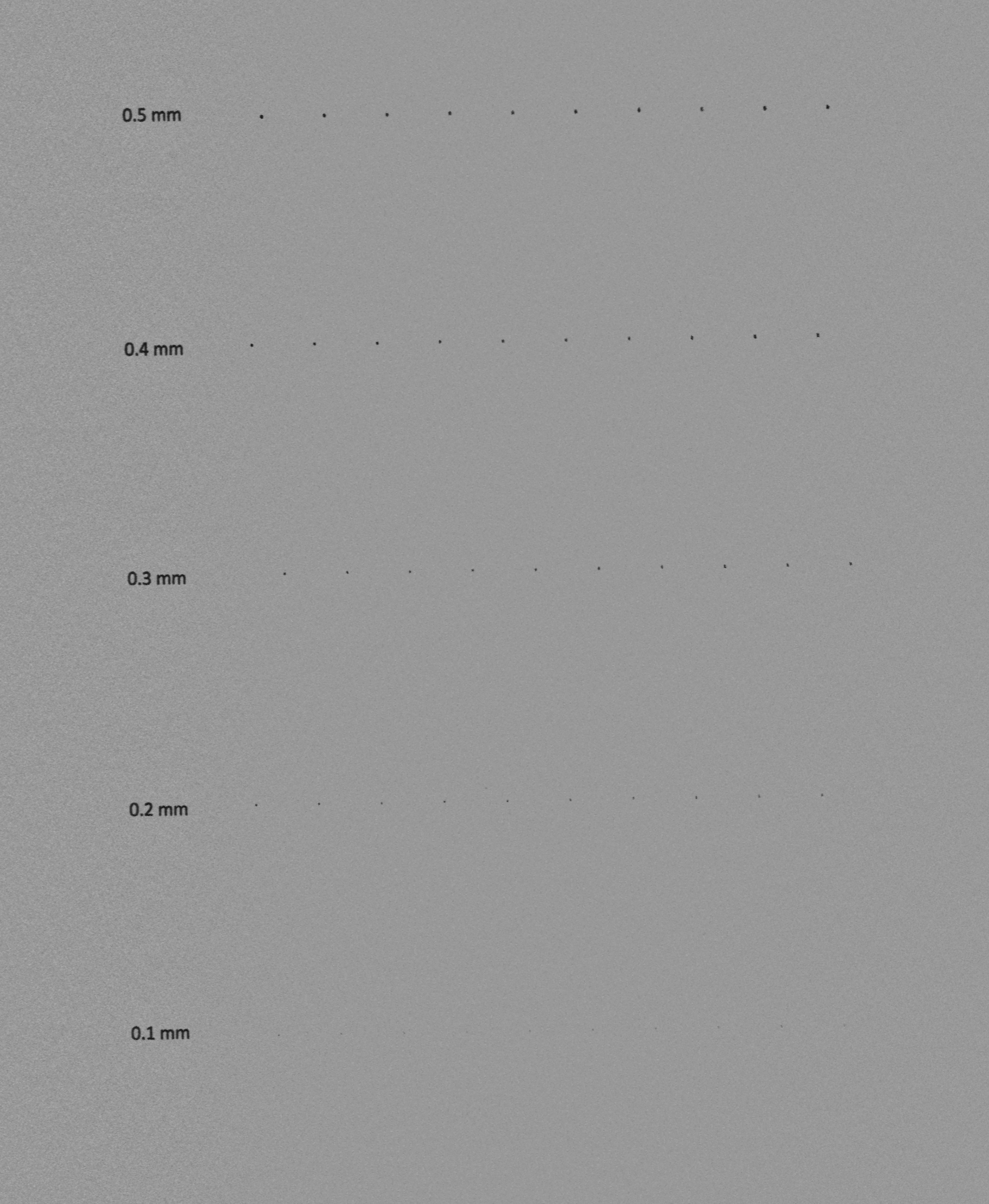

Supplement: Supplementary file 1 [file sensors-25-03426-s001.zip › DotsOnDibond/DotsOnDibond_3_local_median.tif]

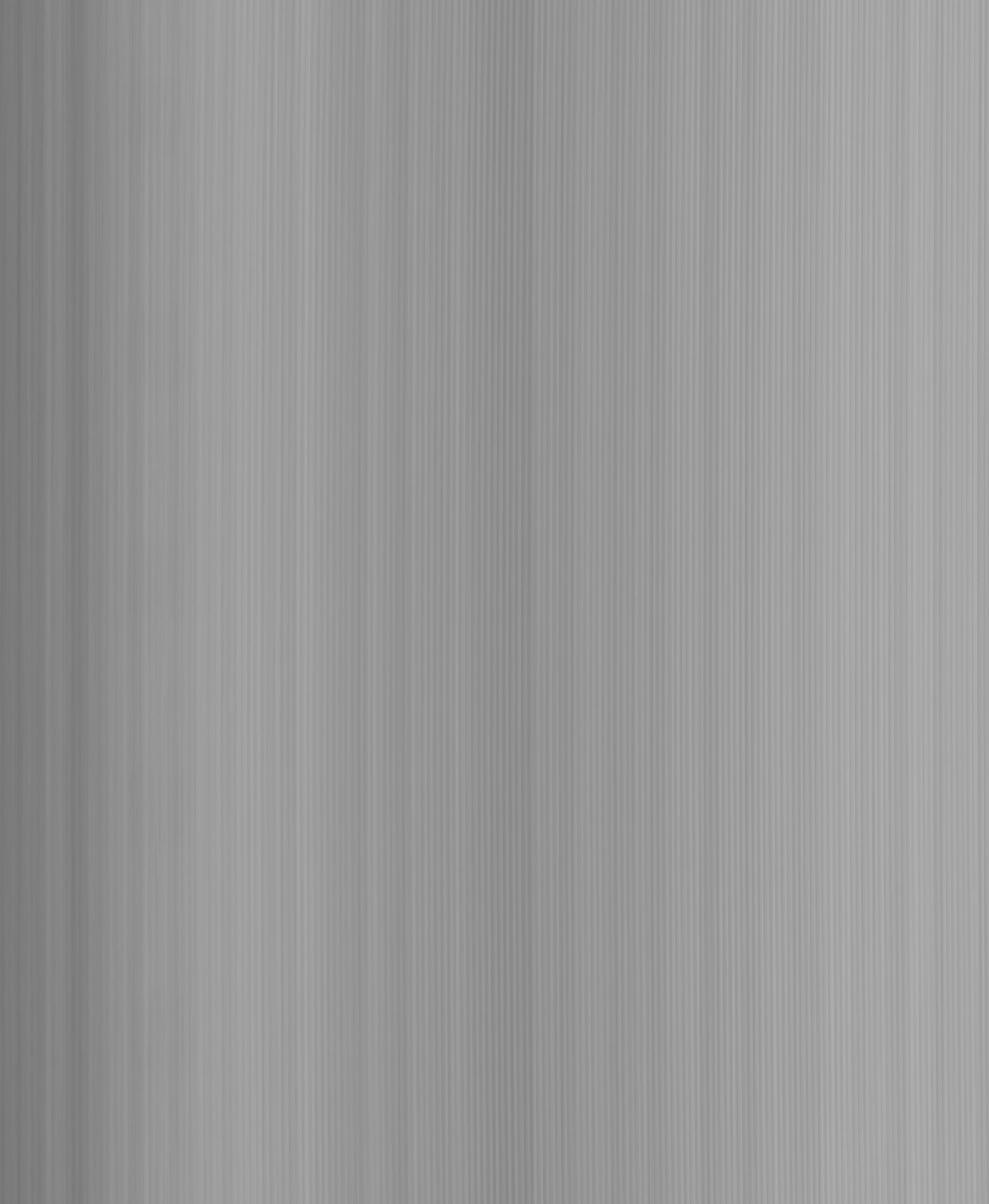

Supplement: Supplementary file 1 [file sensors-25-03426-s001.zip › DotsOnDibond/DotsOnDibond_4_median_image.tif]

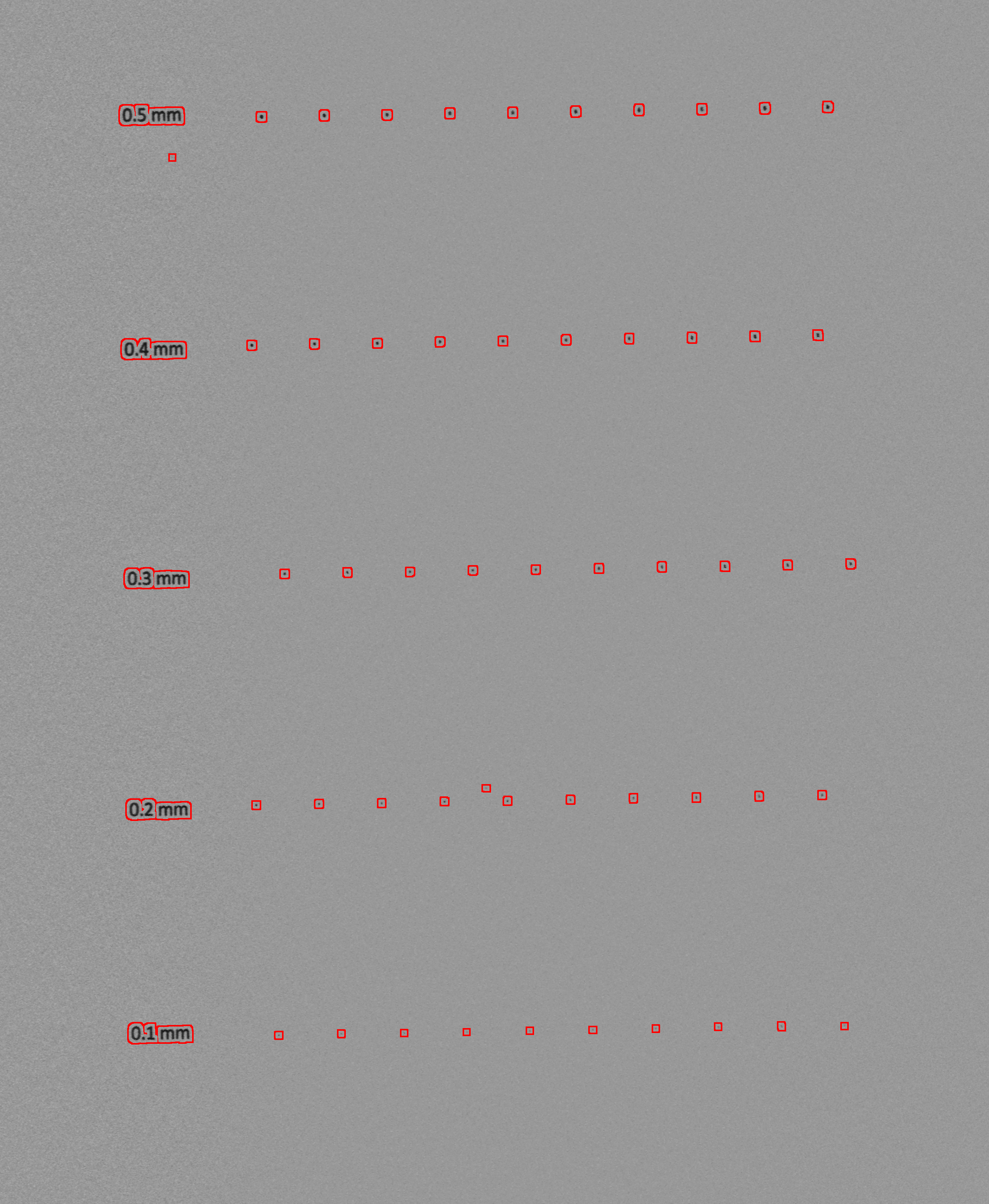

Supplement: Supplementary file 1 [file sensors-25-03426-s001.zip › DotsOnDibond/DotsOnDibond_DETECTION.tif]

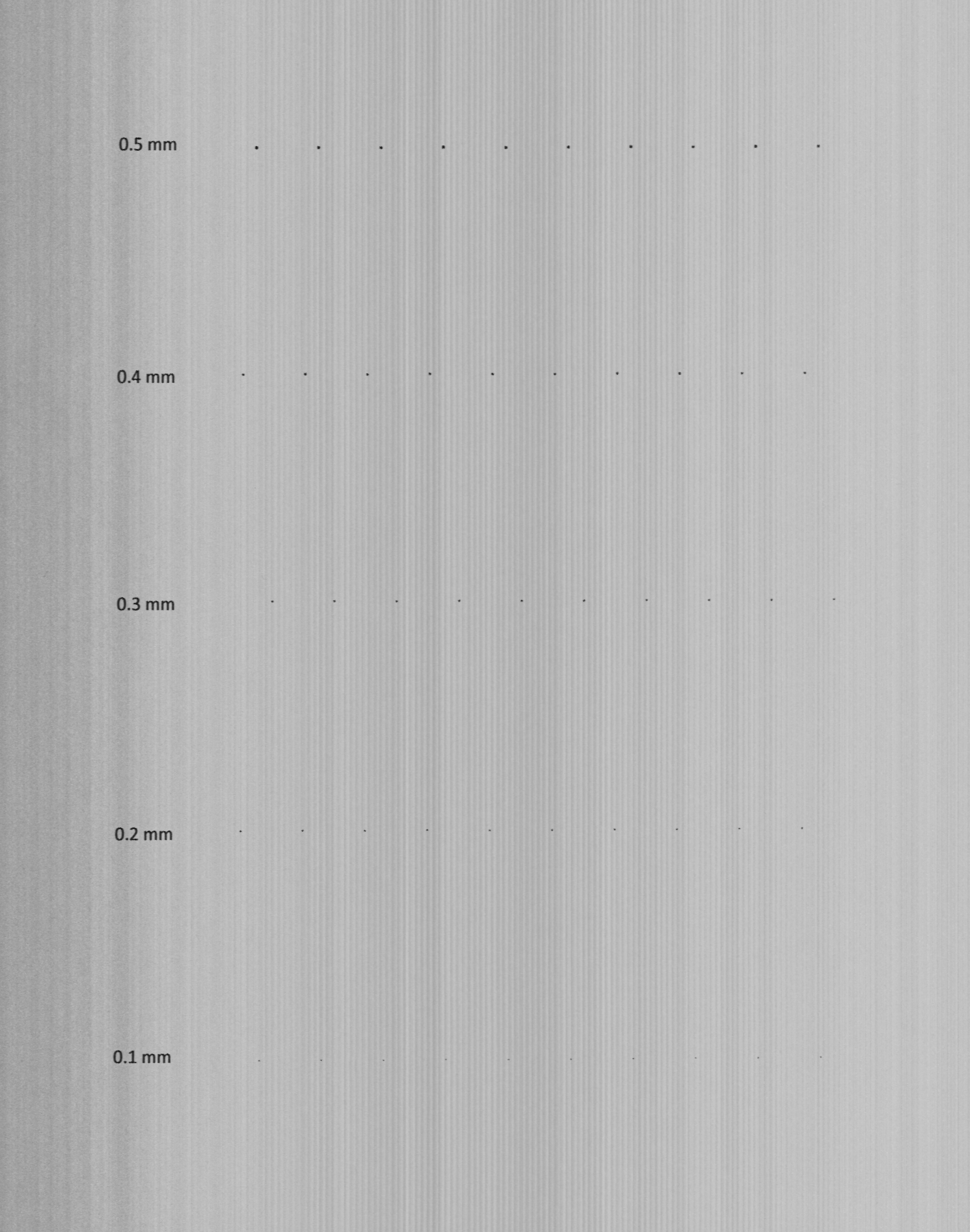

Supplement: Supplementary file 1 [file sensors-25-03426-s001.zip › DotsOnSticker/DotsOnSticker_0_original.tif]

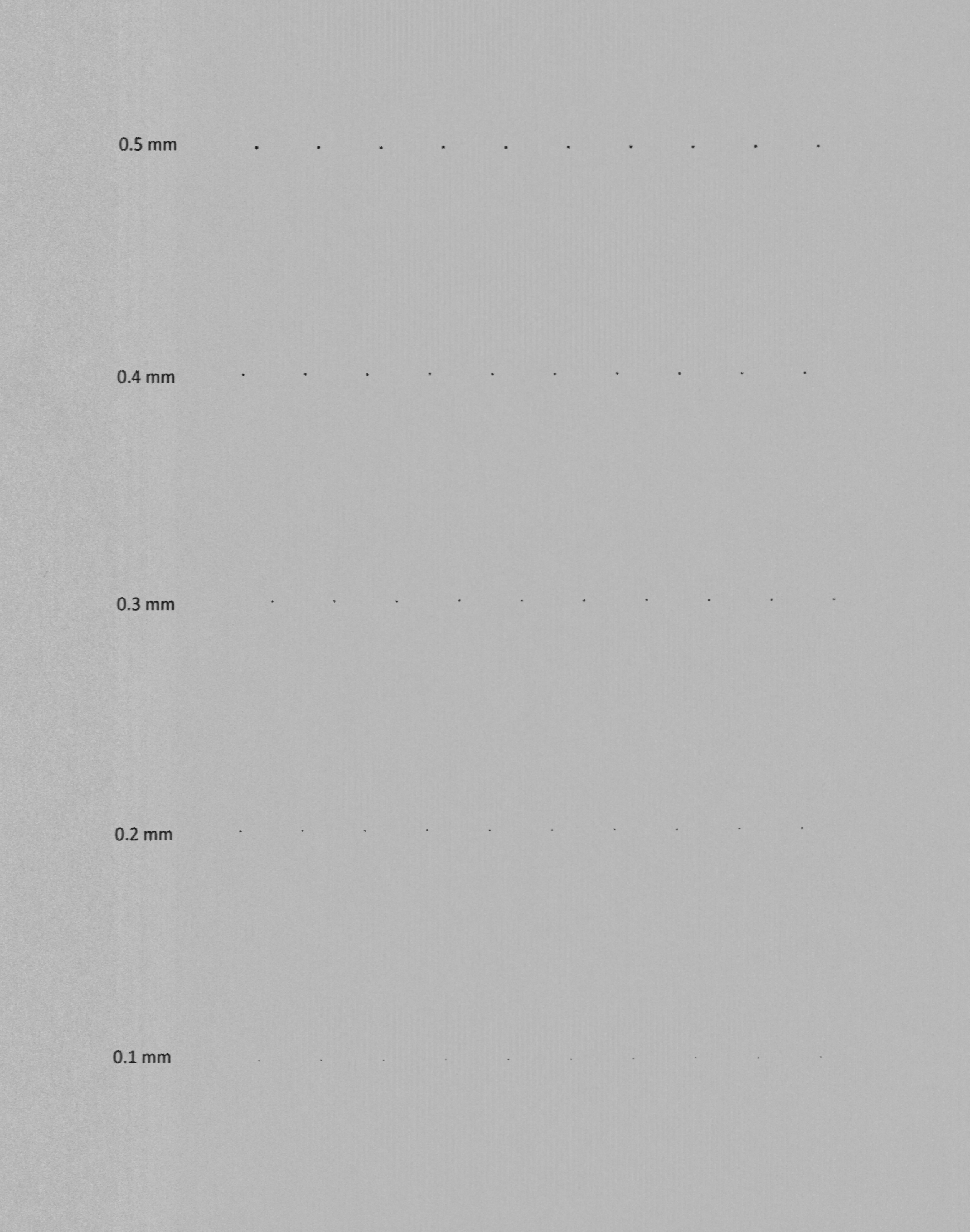

Supplement: Supplementary file 1 [file sensors-25-03426-s001.zip › DotsOnSticker/DotsOnSticker_1_mean.tif]

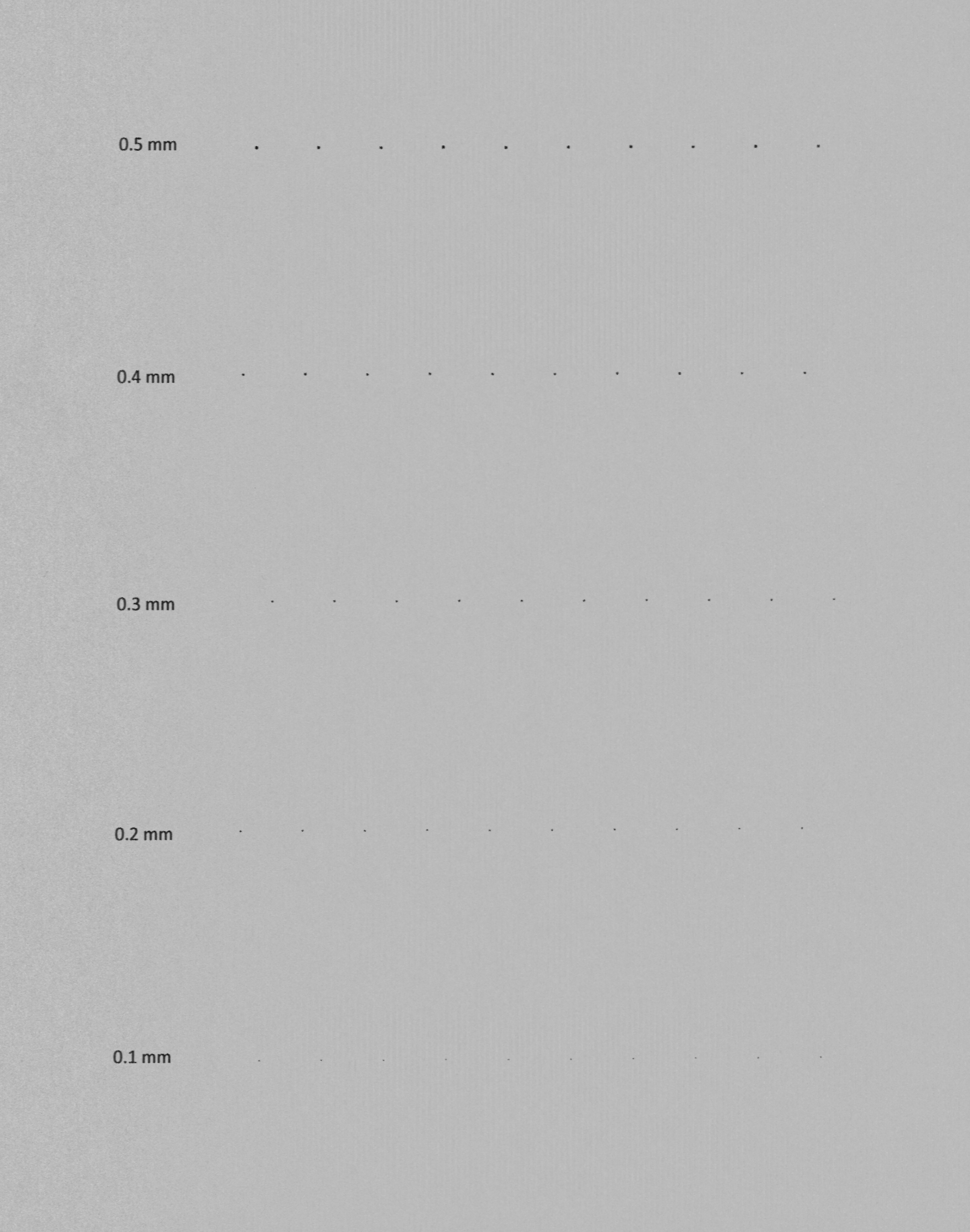

Supplement: Supplementary file 1 [file sensors-25-03426-s001.zip › DotsOnSticker/DotsOnSticker_2_median.tif]

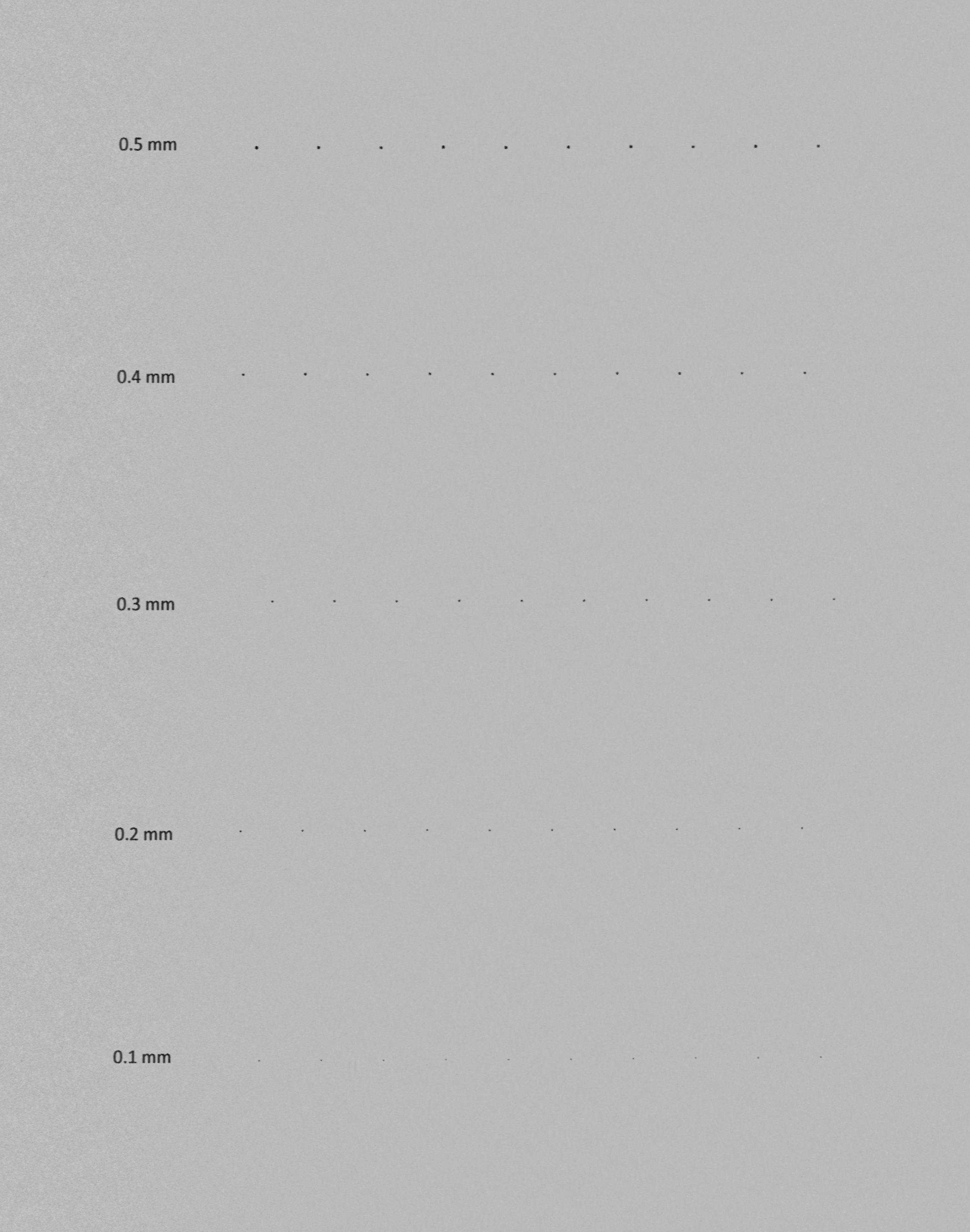

Supplement: Supplementary file 1 [file sensors-25-03426-s001.zip › DotsOnSticker/DotsOnSticker_3_local_median.tif]

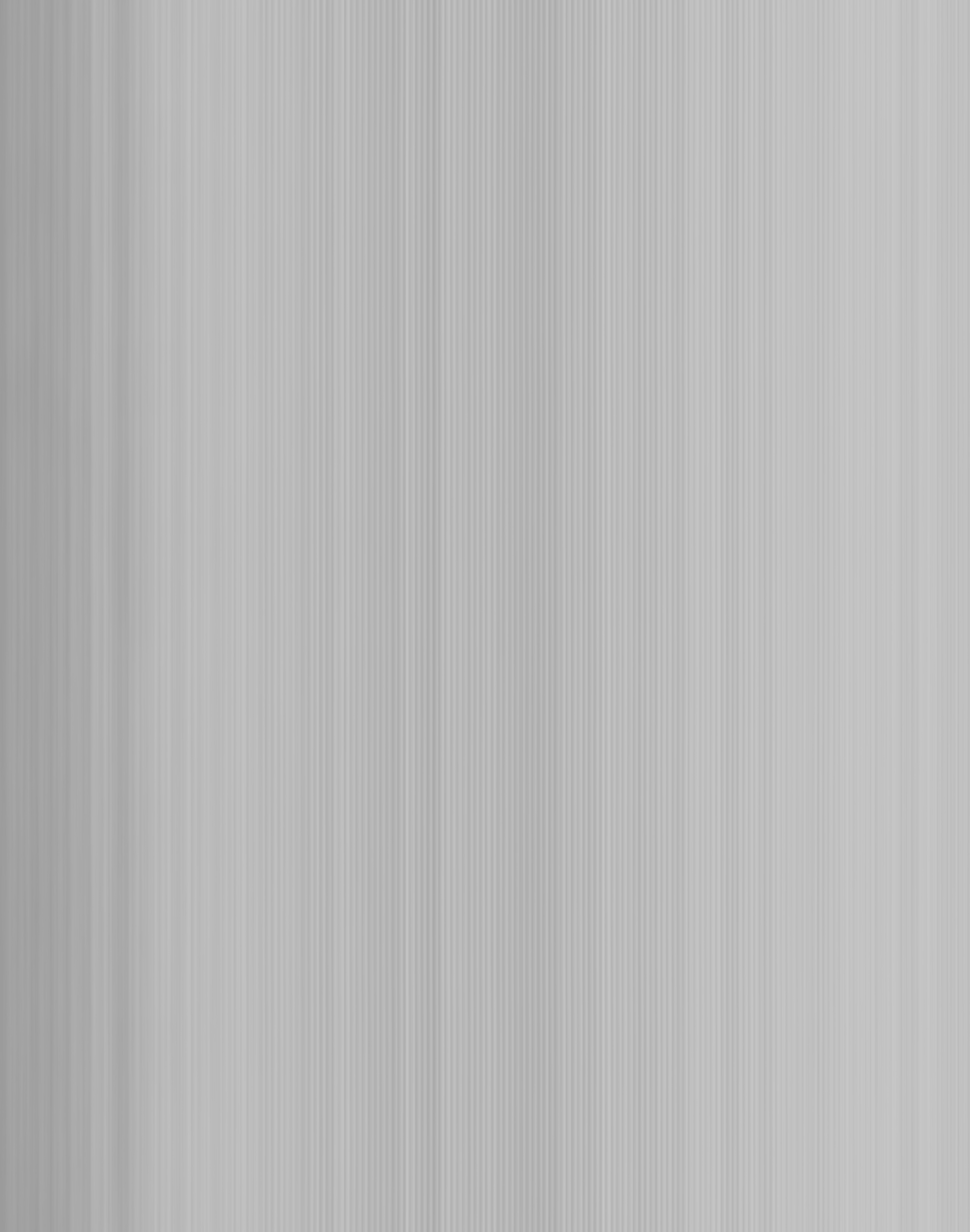

Supplement: Supplementary file 1 [file sensors-25-03426-s001.zip › DotsOnSticker/DotsOnSticker_4_median_image.tif]

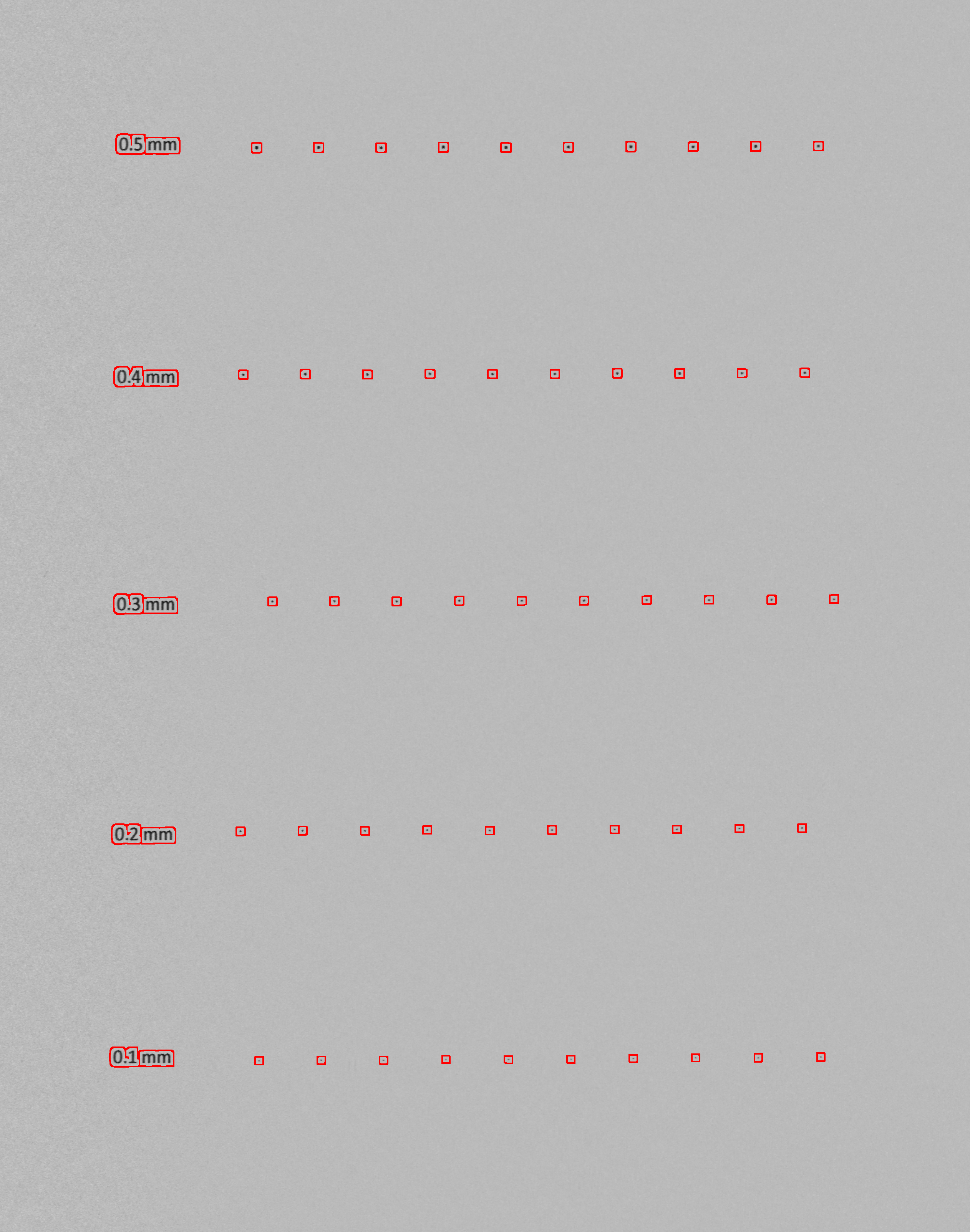

Supplement: Supplementary file 1 [file sensors-25-03426-s001.zip › DotsOnSticker/DotsOnSticker_DETECTION.tif]

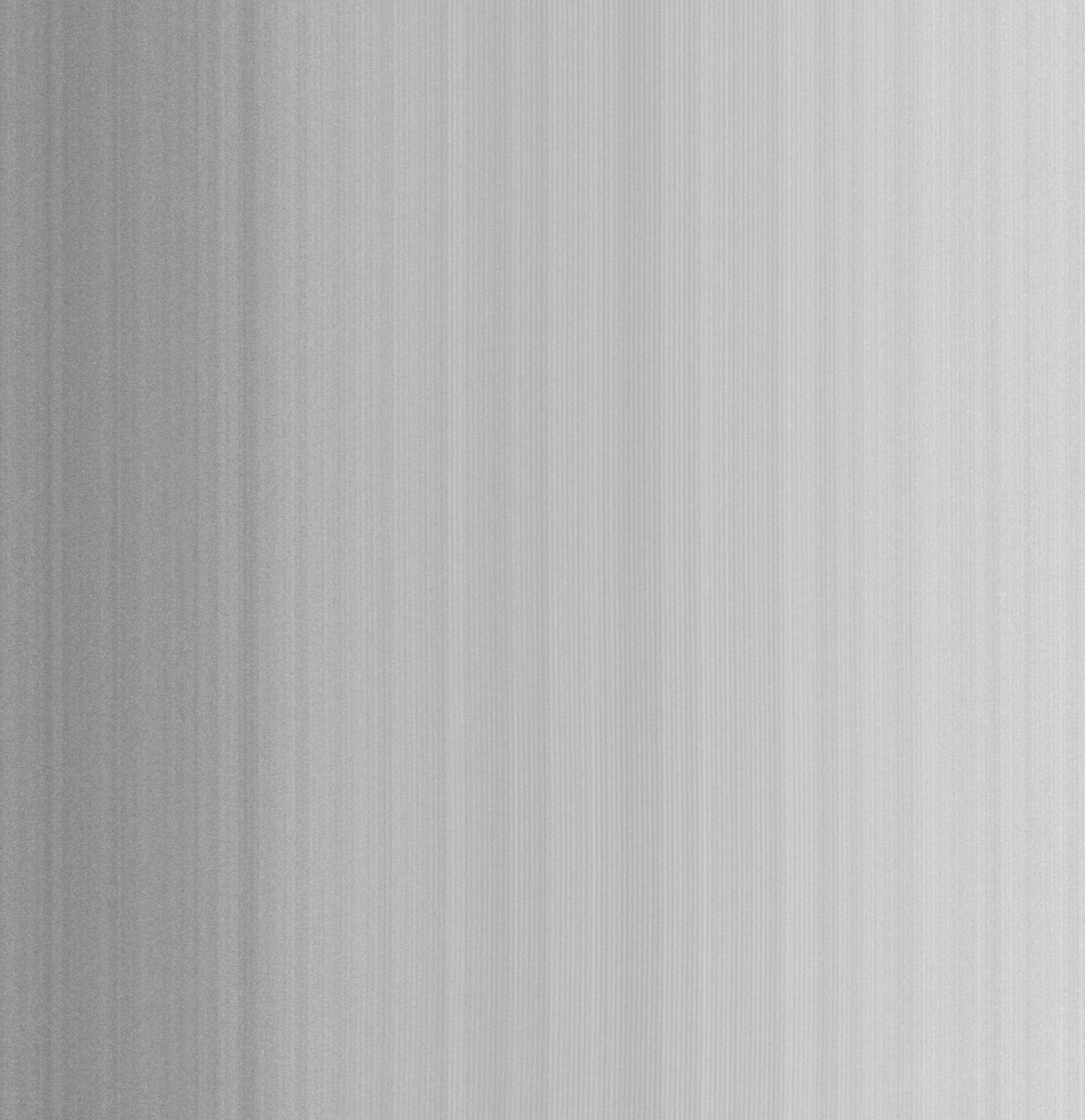

Supplement: Supplementary file 1 [file sensors-25-03426-s001.zip › HDFBoard/HDFBoard_0_original.tif]

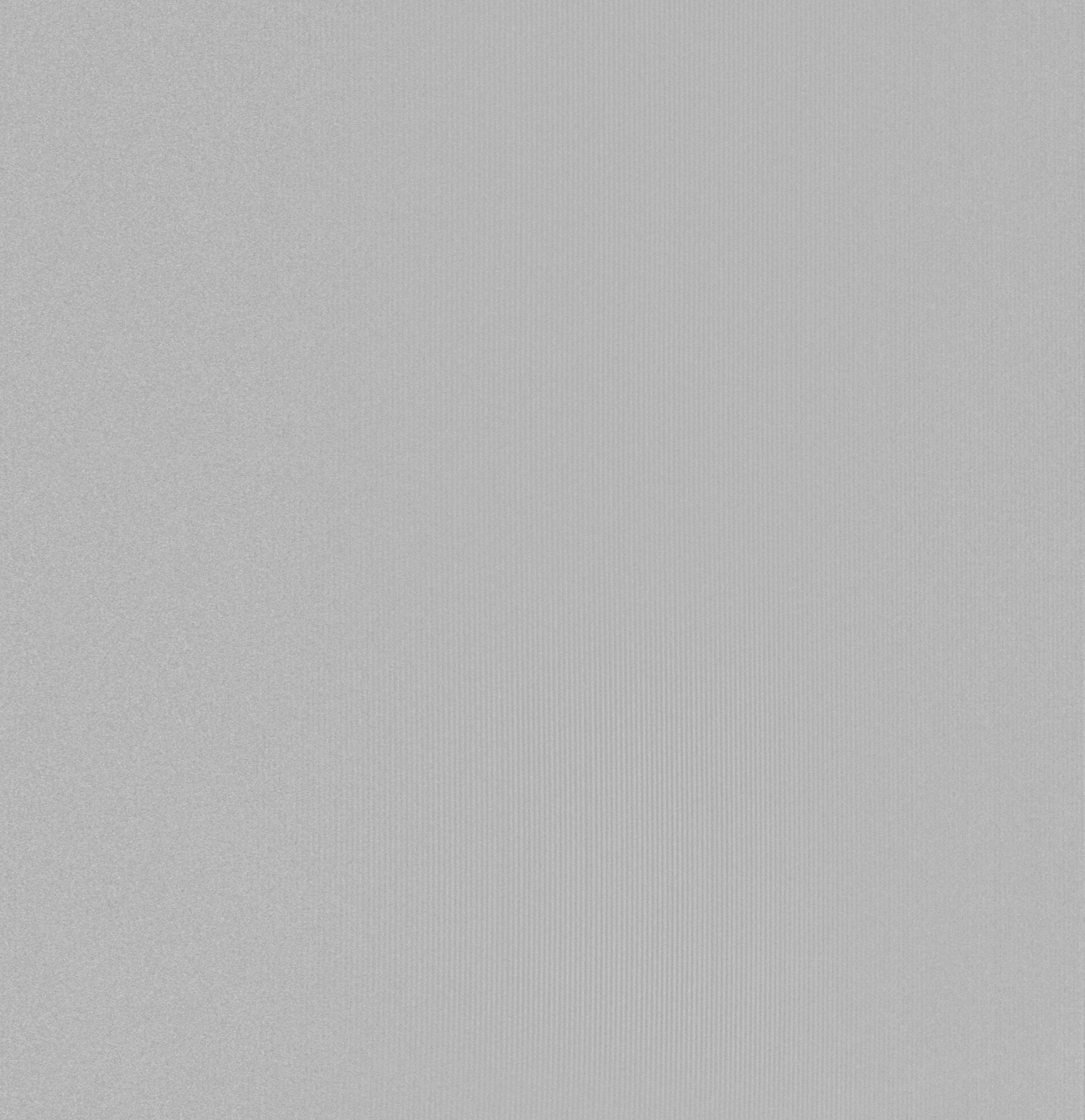

Supplement: Supplementary file 1 [file sensors-25-03426-s001.zip › HDFBoard/HDFBoard_1_mean.tif]

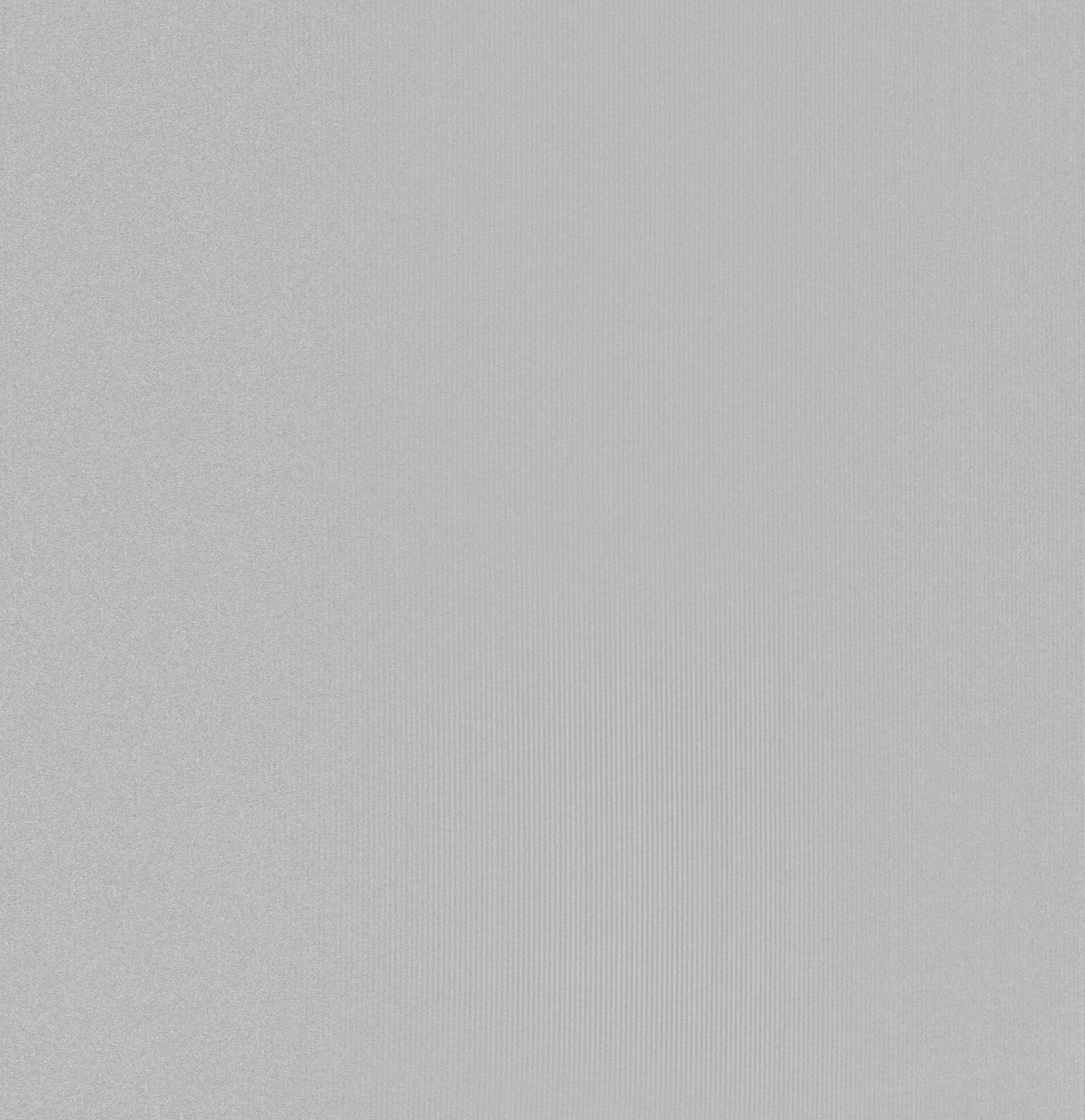

Supplement: Supplementary file 1 [file sensors-25-03426-s001.zip › HDFBoard/HDFBoard_2_median.tif]

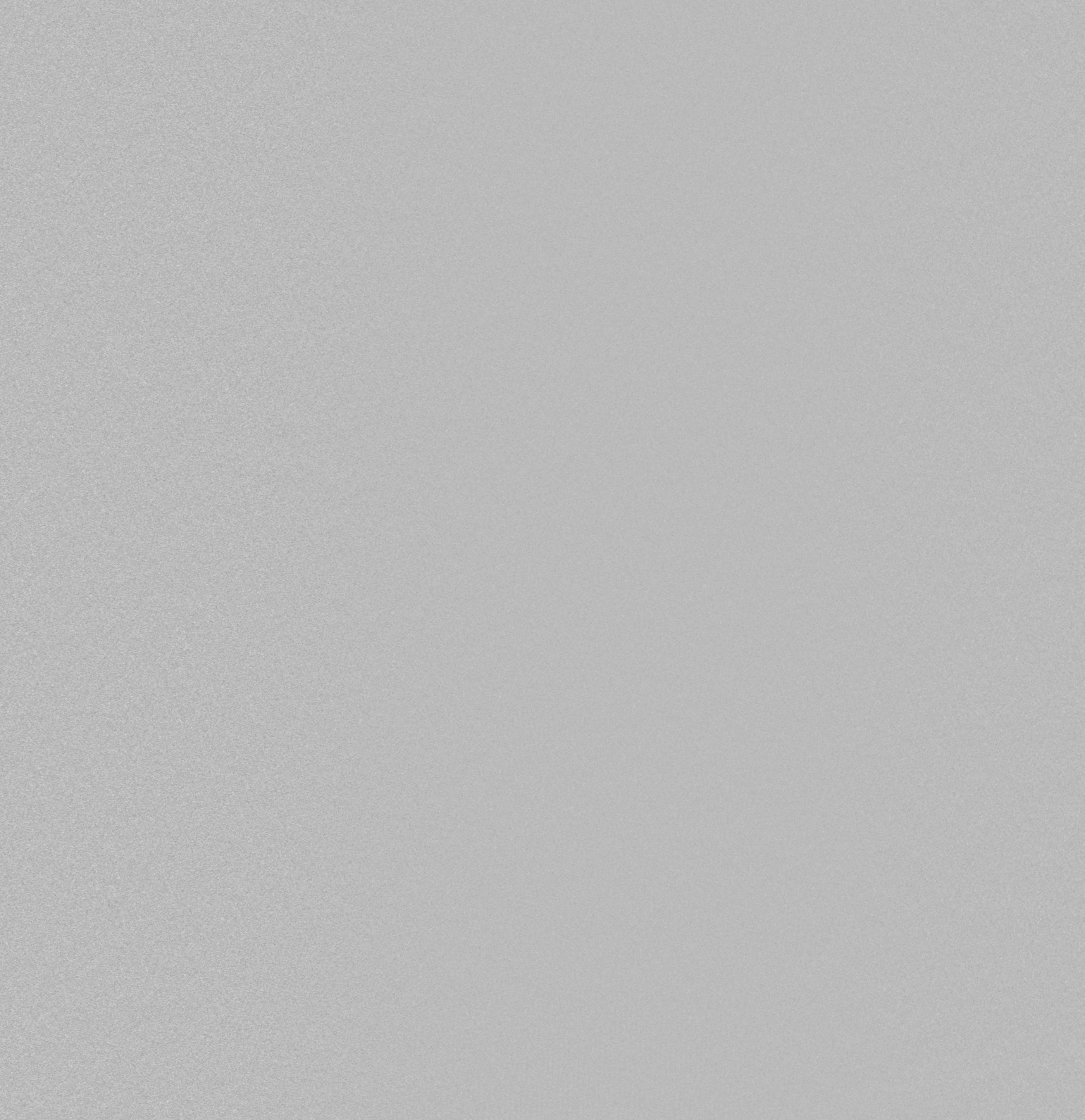

Supplement: Supplementary file 1 [file sensors-25-03426-s001.zip › HDFBoard/HDFBoard_3_local_median.tif]

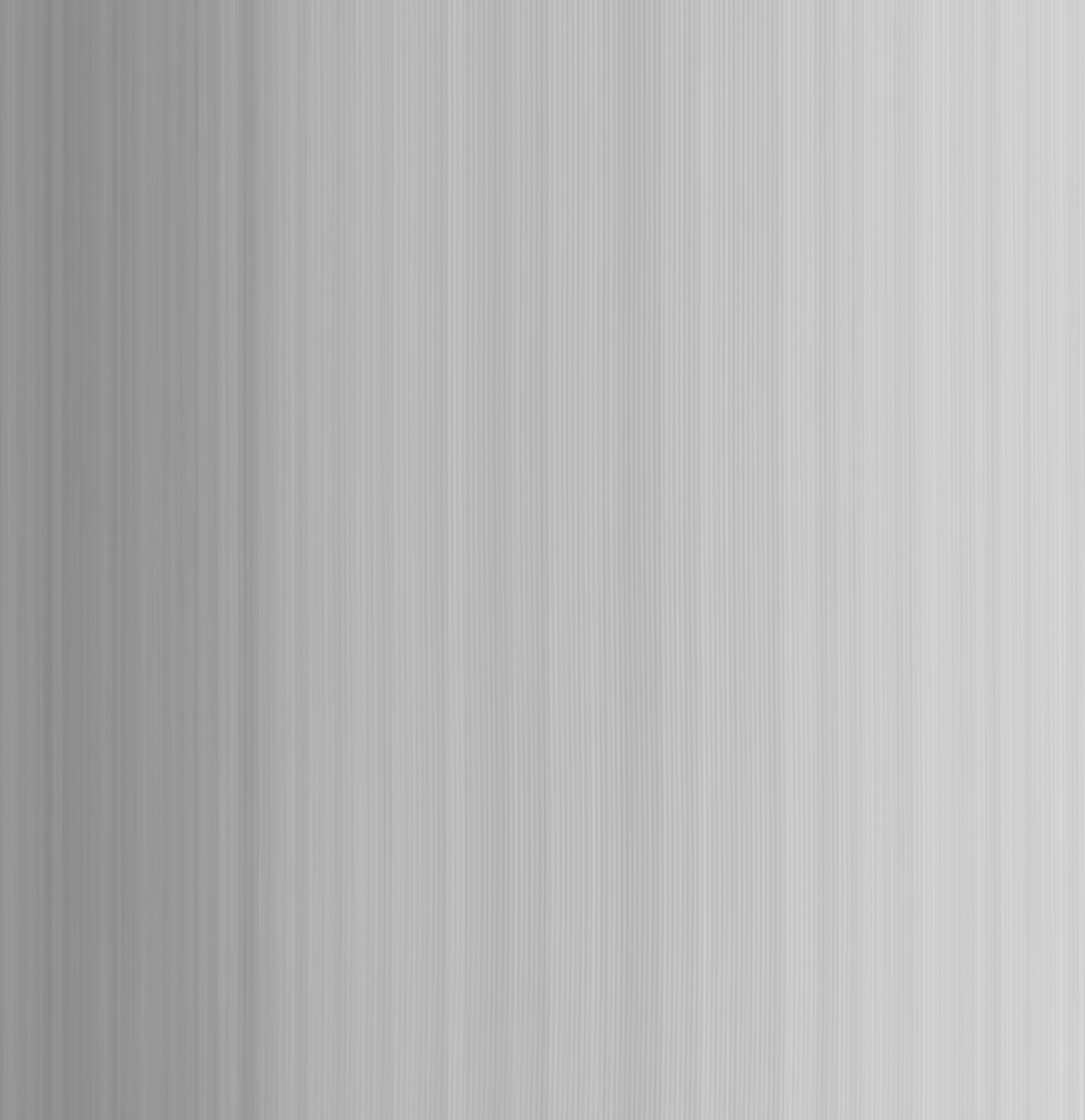

Supplement: Supplementary file 1 [file sensors-25-03426-s001.zip › HDFBoard/HDFBoard_4_median_image.tif]

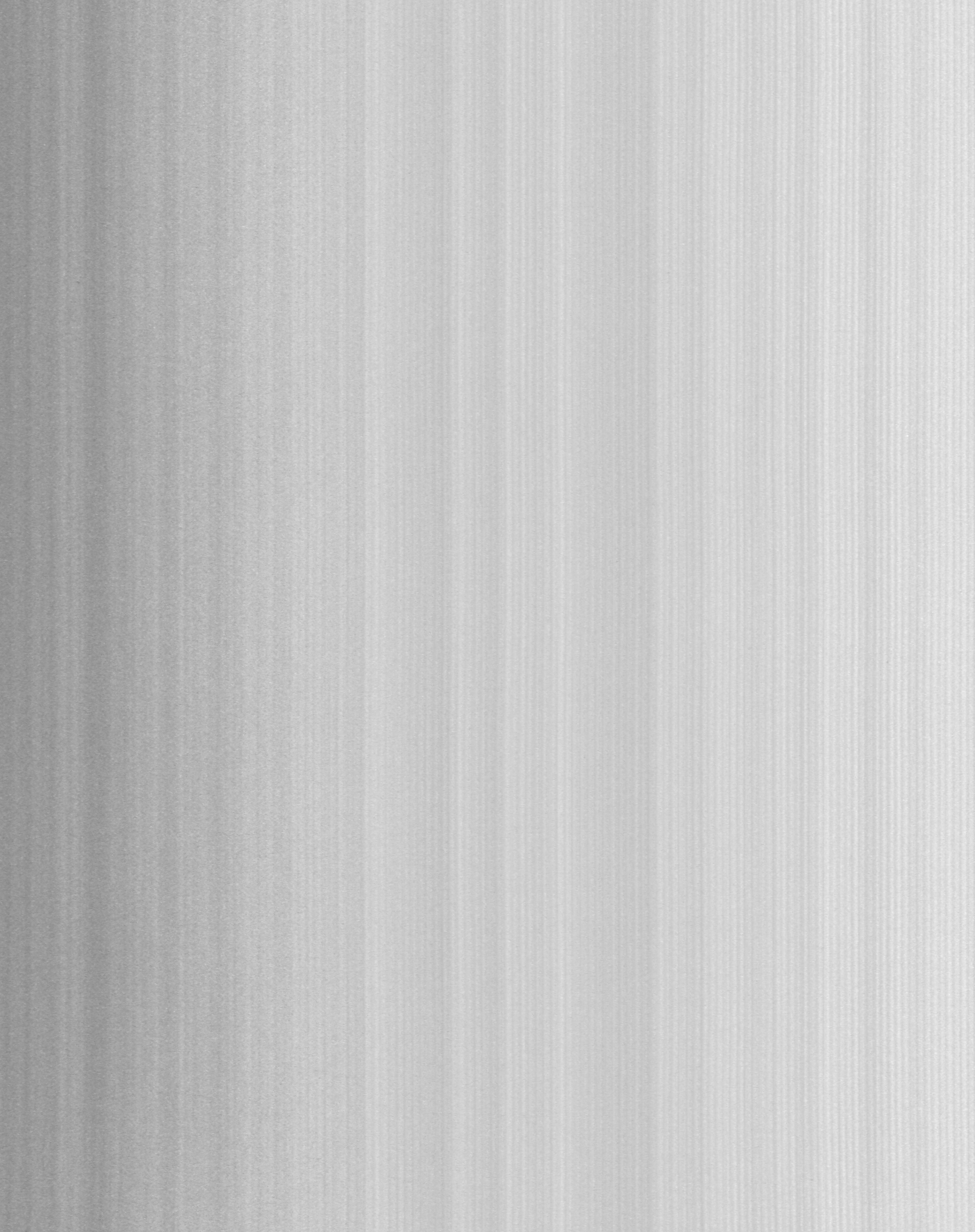

Supplement: Supplementary file 1 [file sensors-25-03426-s001.zip › MousePad/MousePad_0_original.tif]

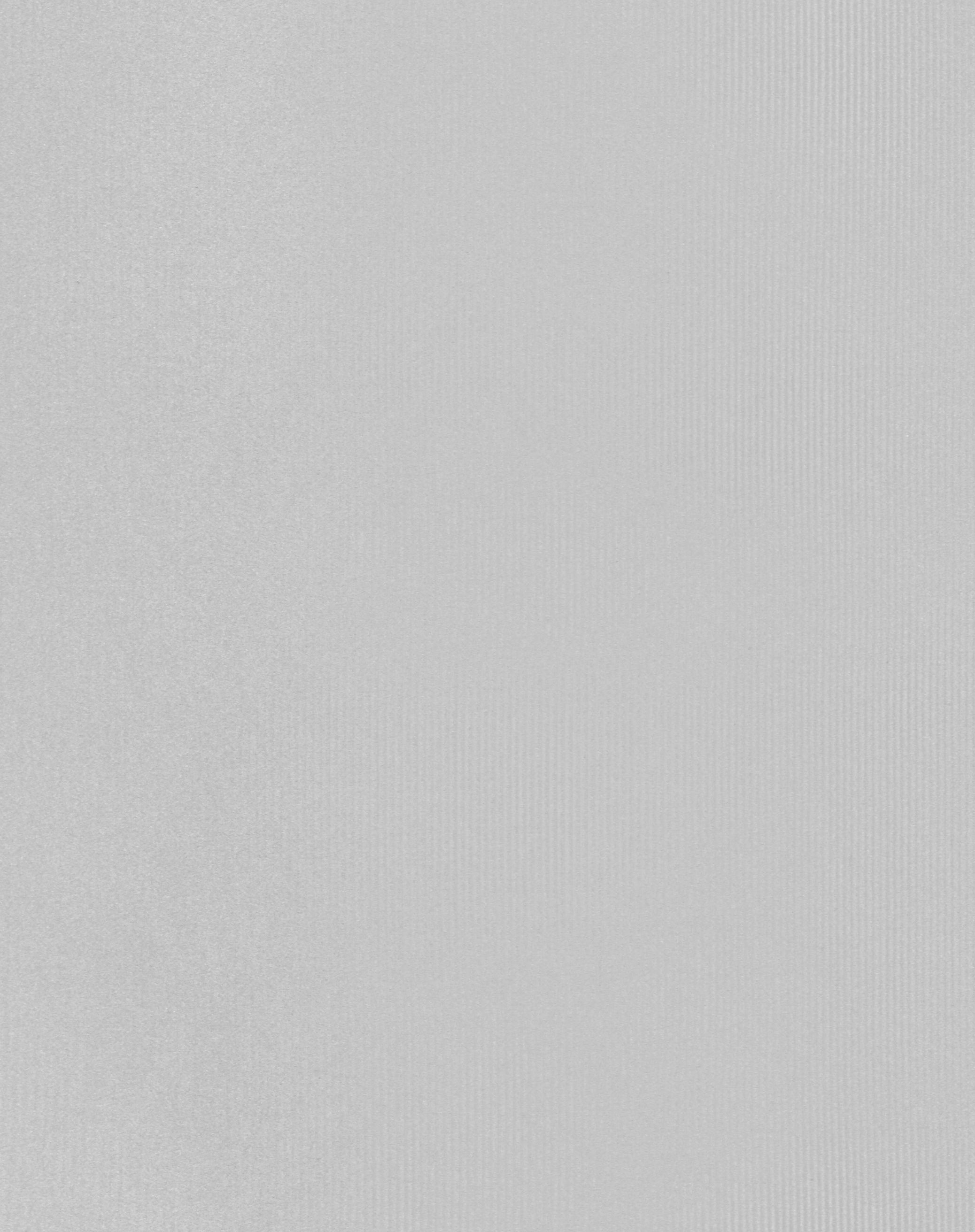

Supplement: Supplementary file 1 [file sensors-25-03426-s001.zip › MousePad/MousePad_1_mean.tif]

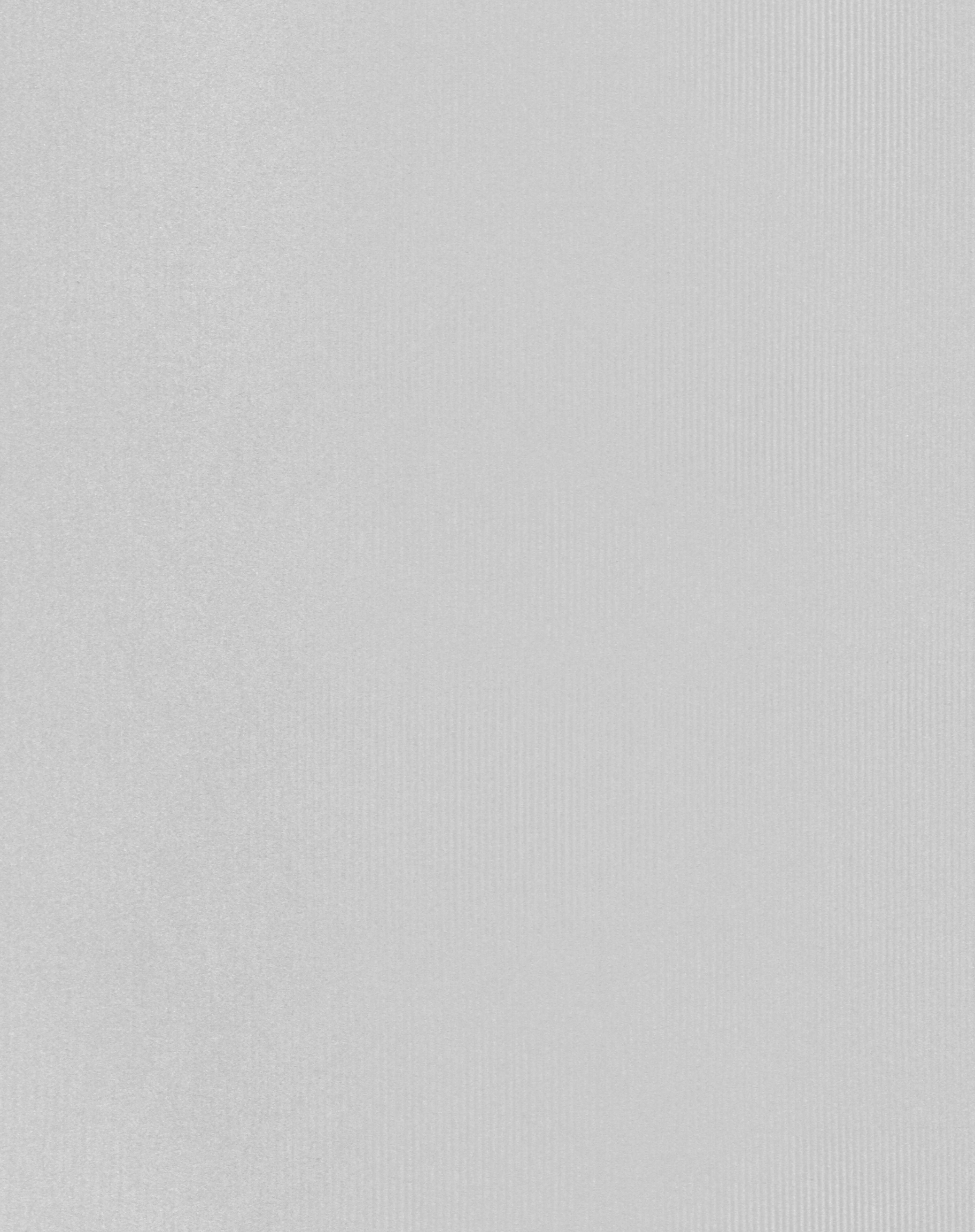

Supplement: Supplementary file 1 [file sensors-25-03426-s001.zip › MousePad/MousePad_2_median.tif]

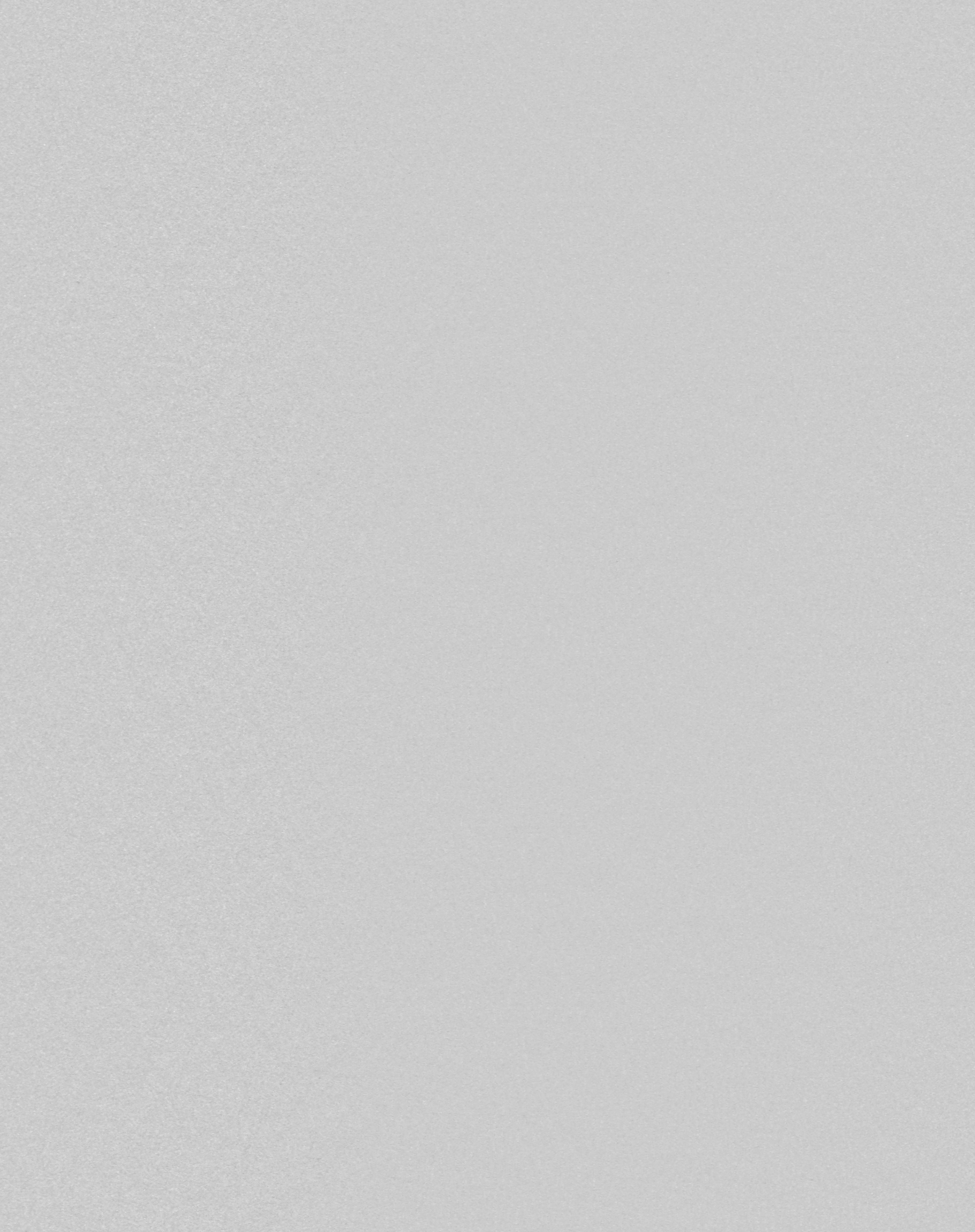

Supplement: Supplementary file 1 [file sensors-25-03426-s001.zip › MousePad/MousePad_3_local_median.tif]

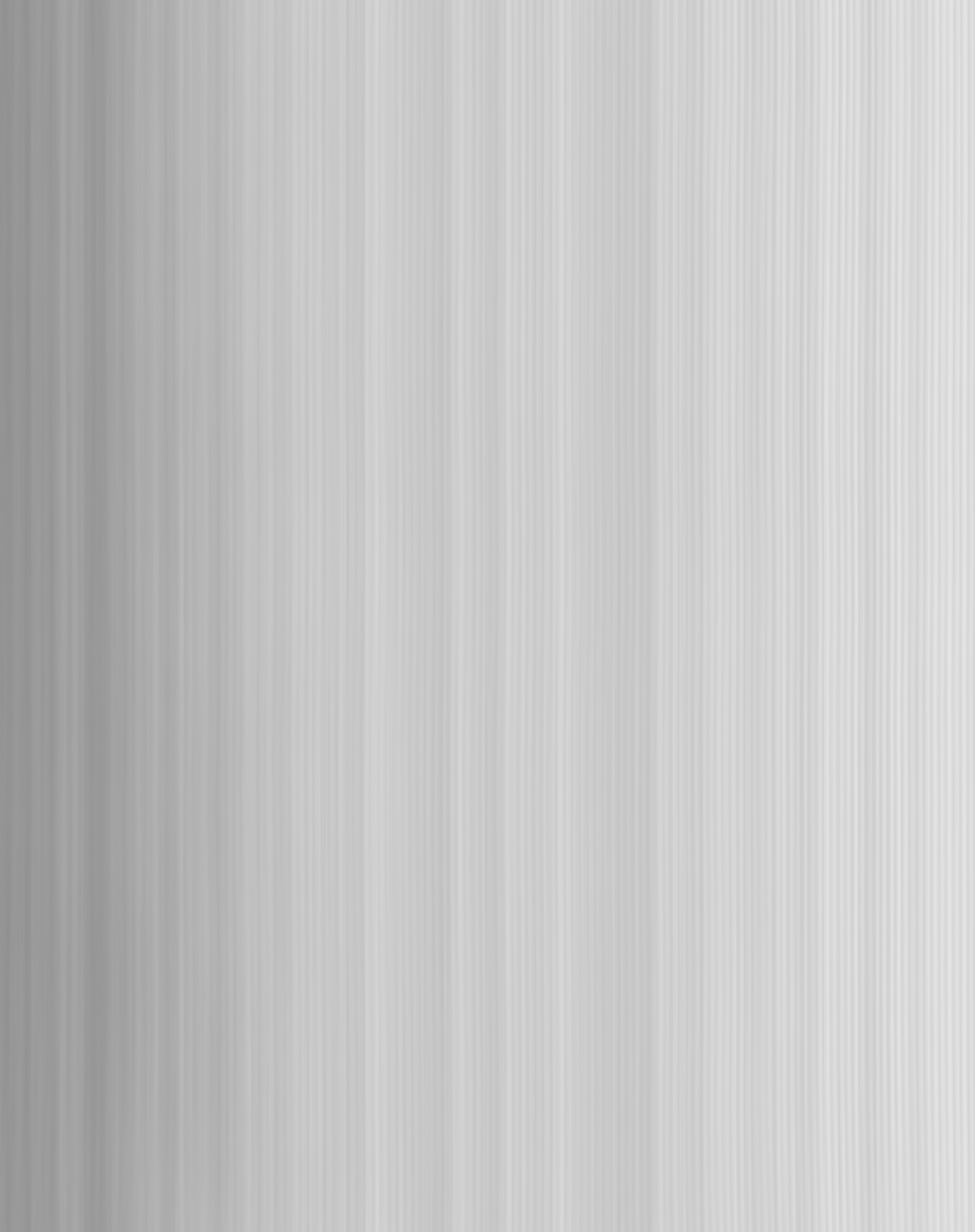

Supplement: Supplementary file 1 [file sensors-25-03426-s001.zip › MousePad/MousePad_4_median_image.tif]

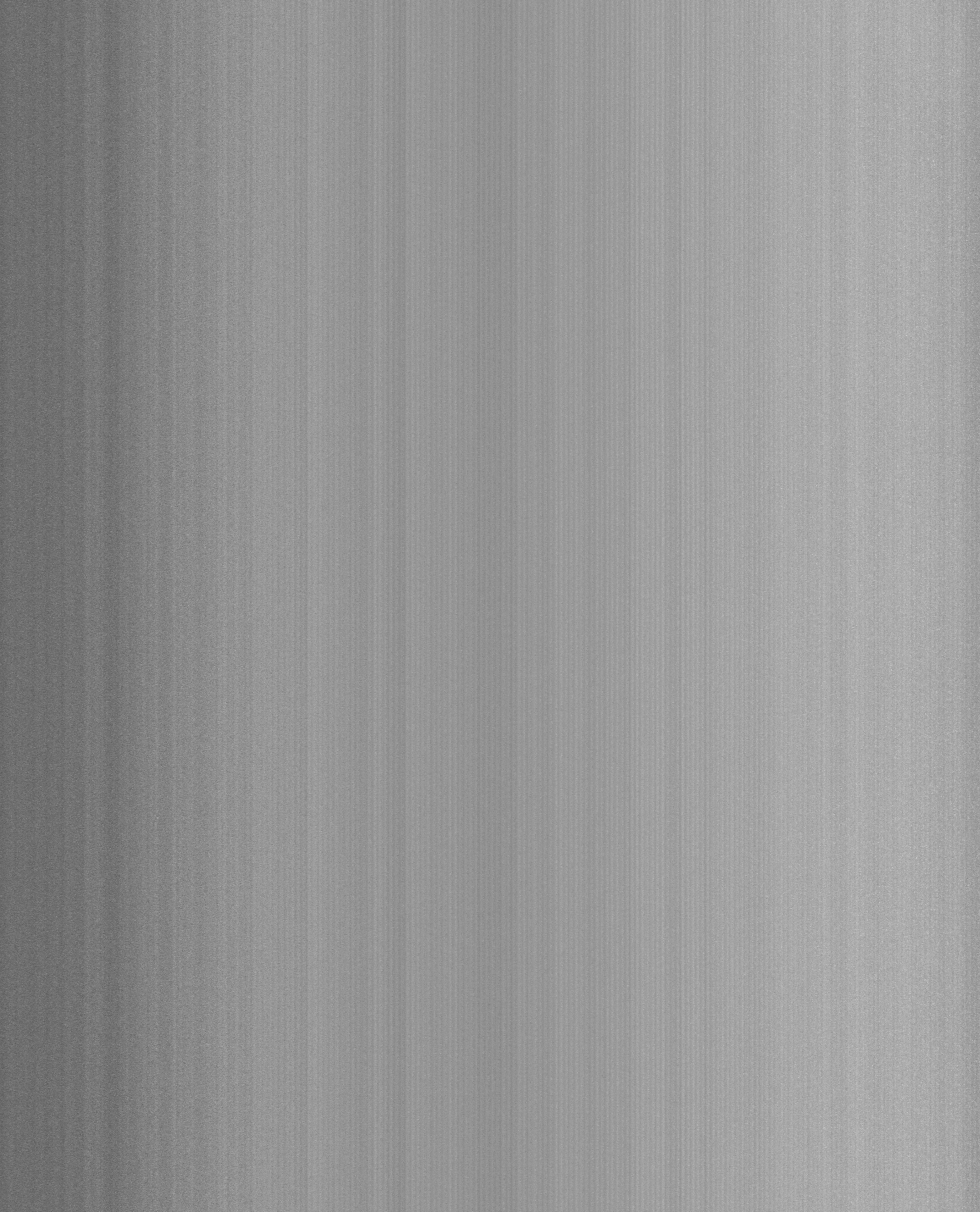

Supplement: Supplementary file 1 [file sensors-25-03426-s001.zip › PaintedFiberboard/PaintedFiberboard_0_original.tif]

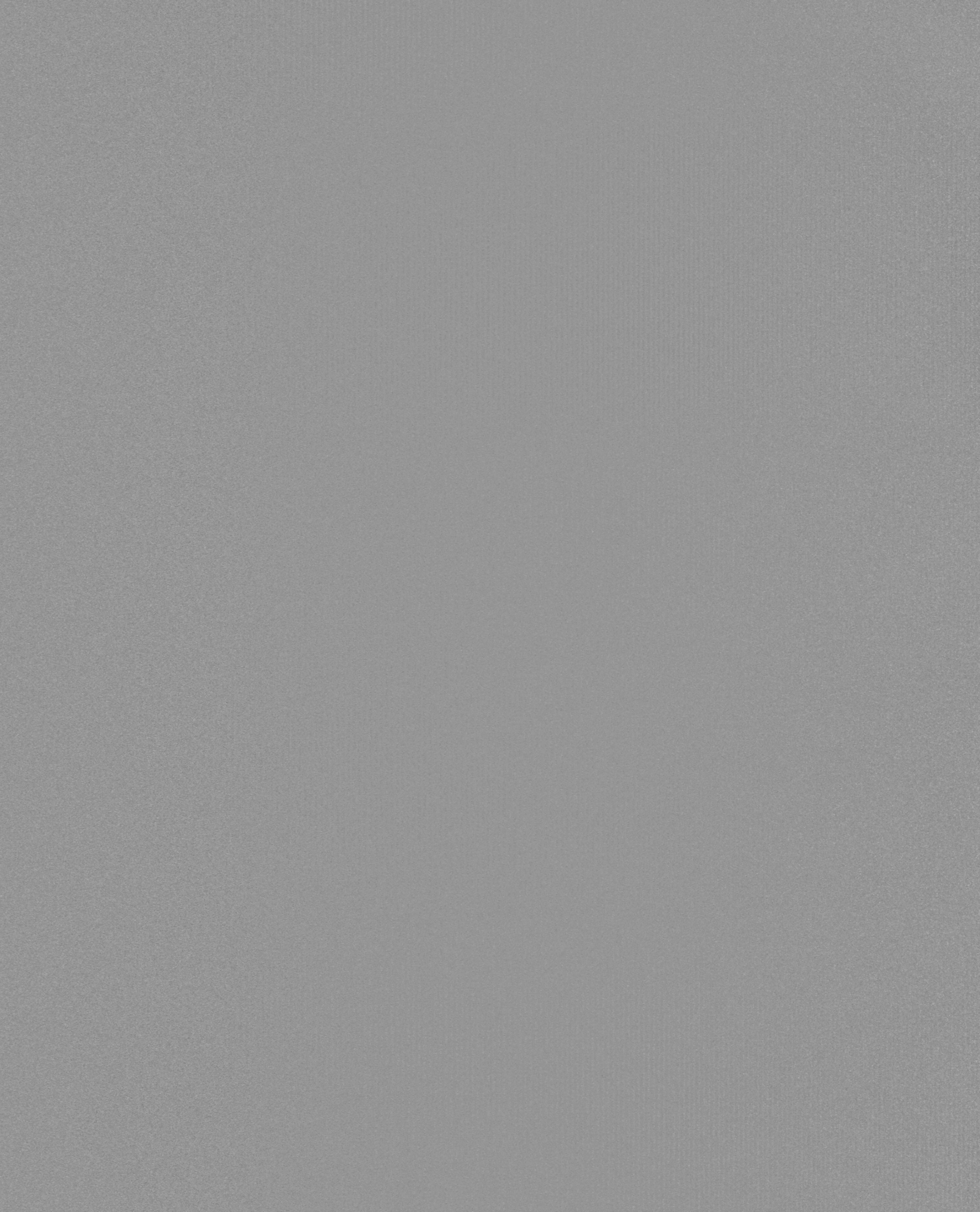

Supplement: Supplementary file 1 [file sensors-25-03426-s001.zip › PaintedFiberboard/PaintedFiberboard_1_mean.tif]

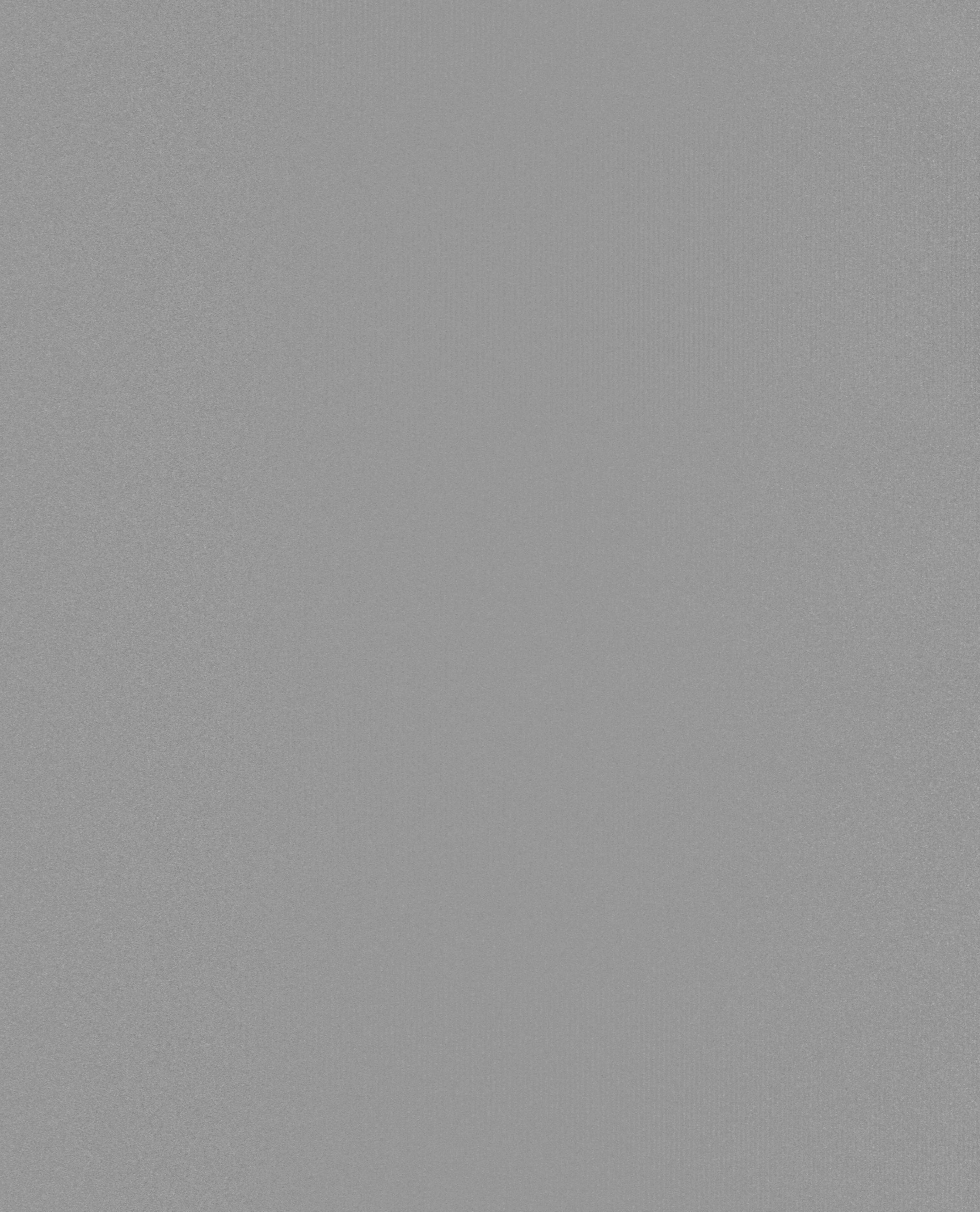

Supplement: Supplementary file 1 [file sensors-25-03426-s001.zip › PaintedFiberboard/PaintedFiberboard_2_median.tif]

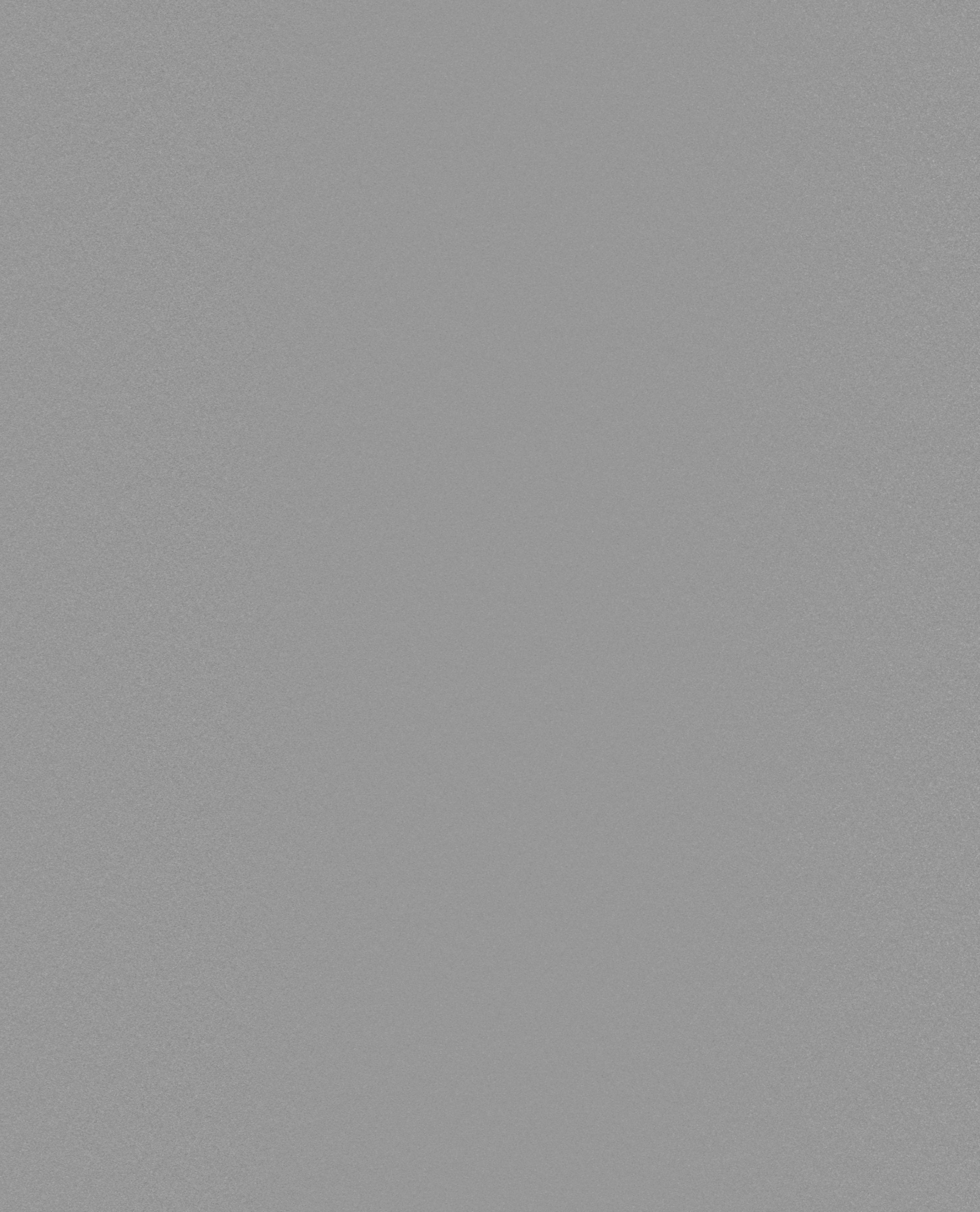

Supplement: Supplementary file 1 [file sensors-25-03426-s001.zip › PaintedFiberboard/PaintedFiberboard_3_local_median.tif]

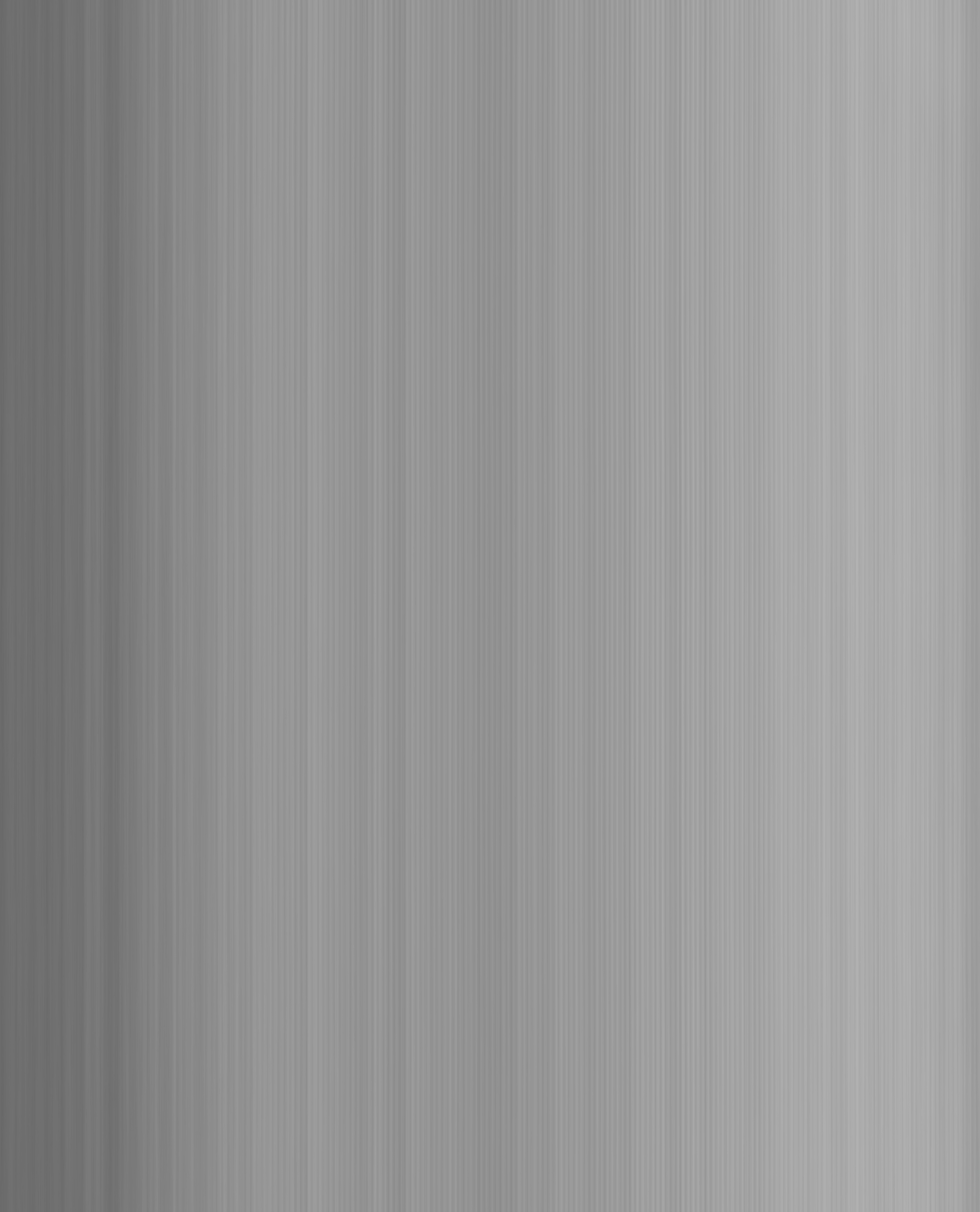

Supplement: Supplementary file 1 [file sensors-25-03426-s001.zip › PaintedFiberboard/PaintedFiberboard_4_median_image.tif]
